# Integrated Chemical and Hazard Assessment of Plastic Pellets from the *Toconao* Spill (Galicia, Spain) Indicates Potential for Environmental Harm

**DOI:** 10.1021/acs.est.5c16166

**Published:** 2026-02-19

**Authors:** Carmen Morales-Caselles, Andy M. Booth, Juan Baztan, Line-Marie Berget, Eric Carmona, Natàlia Corcoll, Hubert Dirven, Montserrat Filella, Daniela Gómez-Martínez, Dorte Herzke, Hege Hjertholm, Annika Jahnke, Per Meyer Jepsen, Azora König Kardgar, Claudia Lorenz, Neema Negi, Elisa Rojo-Nieto, Igor Snapkow, Lisbet Sørensen, Kristian Syberg, Hideshige Takada, Andrew Turner, Bethanie Carney-Almroth

**Affiliations:** † Biology Department, Faculty of Marine and Environmental Sciences, University of Cádiz, Puerto Real, Cádiz 11510, Spain; ‡ Institute of Marine Research (INMAR), University of Cádiz, Puerto Real, Cádiz 11510, Spain; § Department for Climate and Environment, 6298 SINTEF Ocean AS, NO-7465 Trondheim, Norway; ∥ Versailles Saint-Quentin-en-Yvelines University, 78035 Versailles, France; ⊥ Department of Chemical Toxicology, 526795 Norwegian Institute of Public Health, NO-0213 Oslo, Norway; # Department of Exposure Science, 28397 Helmholtz Center for Environmental Research (UFZ), 04318 Leipzig, Germany; ○ Department of Atmospheric Chemistry, Leibniz Institute for Tropospheric Research (TROPOS), 04318 Leipzig, Germany; ∇ Department of Biological and Environmental Sciences, Centre for Future Chemical Risk Assessment and Management Strategies (FRAM), 3570 University of Gothenburg, 40530 Gothenburg, Sweden; ☆ Department F.-A. Forel, 27212 University of Geneva, 1205 Geneva, Switzerland; ◮ Division of Climate and Environmental Health, 25563 Norwegian Institute of Public Health, NO-0213 Oslo, Norway; ◀ NILU, NO-9296 Tromsø, Norway; ⊡ Institute for Environmental Research, RWTH Aachen University, 52074 Aachen, Germany; ⬠ Department of Science and Environment, 6976 Roskilde University, 4000 Roskilde, Denmark; ⬣ Norwegian University of Science and Technology (NTNU), Department of Chemistry, NO-7491 Trondheim, Norway; ◓ Laboratory of Organic Geochemistry, Tokyo University of Agriculture and Technology, 183-8509 Tokyo, Japan; ◊ School of Geography, Earth and Environmental Sciences, Plymouth University, PL4 8AA Plymouth, United Kingdom

**Keywords:** Nurdles, Additives, Ecotoxicity, Nonintentionally Added Substances, Microplastics, Marine Environmental Policy, Human PBMCs

## Abstract

Plastic pellet spills are a major source of microplastic pollution, and pellets are found on beaches worldwide. However, the potential environmental impacts of these spills remain poorly understood. In December 2023, approximately 25,000 kg of polyethylene pellets containing high concentrations of the additive Tinuvin UV-622 were spilled during a shipping accident off the northern coast of Portugal. Pellets collected from an affected beach located in Galicia, Spain, along with solvent extracts and aqueous leachates, were subjected to both target and nontarget chemical analyses and tested in a battery of toxicity assays including a green microalga (*Raphidocelis subcapitata*), a marine copepod (*Apocyclops royi*), a fish model (*Danio rerio*), and a human cell line. Chemical screening identified on the order of 50 chemical substances in addition to Tinuvin UV-622, including a range of known plastic additives and nonintentionally added substances (NIAS). Toxicity assays revealed significant growth inhibition and stress-induced cell aggregation in *R. subcapitata* and acute toxicity causing immobilization in copepods, which could have potential implications in the environment via the disruption of primary producers and food web dynamics. In contrast, zebrafish embryos showed no significant developmental effects, while human cells exhibited modest, time-dependent reductions in viability. Our findings underscore the complex chemical burden associated with pellet spills and stress the need for policies and regulations to prevent them, reinforcing the importance of applying the precautionary principle in managing the environmental risks linked to plastic pellet production, transport, and accidental release.

## Graphical abstract

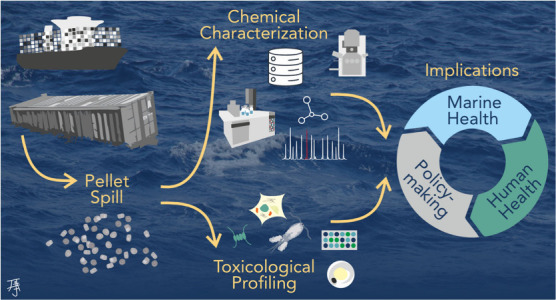

## 1 Introduction

Plastic pellet loss is the third largest source of unintentionally released microplastics and the second largest source of primary microplastics, with ∼450,000 tons contaminating ecosystems annually.^1^ Pellet distribution is generally associated with onshore sources like industrial sites, ports, and cities, but they also reach remote coastlines via maritime transport losses, ship accidents, and long-distance dispersal by ocean currents.^2^ Low density pellets, or nurdles, can float and travel vast distances via ocean fluxes and marine organisms,^1,3,4^ with beaches and coastal environments acting as major global accumulation zones.^5,6^ In contrast, higher density pellets or biofouled pellets tend to sink and accumulate on the seafloor.^7^

Pellets can contain hazardous chemicals and may act as both sources and vectors of toxic substances in marine environments.^8,9^ Due to their small size, pellets are easily ingested by seabirds and marine organisms, representing up to 36% of ingested plastic in some cases.^10^ Pellet ingestion can lead to physical damage, behavioral changes, and exposure to endocrine-disrupting and immune-altering chemicals, posing significant risks to the development, health, and reproduction of marine organisms.^11^

Since 2010, at least 14 major pellet spills from shipping incidents have been documented (Table S1), although the true number is likely higher due to the lack of mandatory reporting. In 2022 alone, an estimated 230,000 tons of pellets were lost globally,^12^ with the 2021 *X-Press Pearl* disaster off Sri Lanka remaining the largest recorded pellet spill.^2^ A more recent spill occurred on December 8, 2023, when the *Toconao* vessel lost six containers near northern Portugal, one of them carrying a thousand 25 kg sacks of polyethylene (PE) pellets. The pellet sacks that a few days later washed ashore in Galicia (Spain) were labeled as “*Bedeko Europe Code UV3000 Stabilizator UV/UV Stabilizer*” and, according to material safety data sheets provided by the manufacturer to Maersk, the shipping company, were composed of 87–90% PE and 10–13% of the UV stabilizer ‘Tinuvin UV-622’, a hindered amine light stabilizer known to be harmful to aquatic life with long-lasting effects.^13^ The possible presence of other additives or other plastic-associated chemicals in the pellets could not be inferred from the product information available.

The first pellets reached Espiñeirido Beach in Galicia, Spain, on December 13, 2023, but media coverage only began in January 2024.^14,15^ Although the total volume of pellets spilled (approximately 26.3 tons) was smaller than that of the X-Press Pearl disaster (1,680 tons), the nature of the spilled material—preproduction plastic pellets—was similar in both cases. Early investigations found pellets on 94% of 31 surveyed beaches in Galicia, Asturias in Spain, and northern Portugal, with up to 40.3 pellets per kg of sand.^16^ Nearly half of the pellets were traced to the *Toconao* spill based on their physicochemical characteristics.

Subsequent chemical analyses of pellets collected on Nemiña Beach (Galicia, Spain) confirmed PE as the primary component, along with substantial levels of Tinuvin 622.^17^ Additional additives such as Tinuvin 123, Tinuvin 120, Sanol LS 770, and other industrial chemicals including 4-methylbenzyl alcohol, dimethyl succinate, and calcium stearate were detected in smaller quantities.^17^ Recent studies suggest the *Toconao* pellet spill may have impacted migratory glass eels in Spanish estuaries, introducing white PE particles and causing elevated microplastic accumulation in these sensitive organisms.^18^ This recent spill along the Galician coast highlights the persistent and deleterious impact of anthropogenic microplastic pollution in marine environments.

In this study, the environmental hazards posed by pellets collected from beaches in northern Spain following the *Toconao* spill were investigated in the laboratory. Multiple analytical techniques were used to study both intentionally added substances (e.g., additives) and nonintentionally added substances (NIAS) in pellets. Aqueous leachates were generated from the collected pellets, chemically characterized, and evaluated for their toxicological effects using a range of model organisms representing different trophic levels. By combining chemical and bioanalytical profiling, the study aims to advance understanding of the environmental hazards posed by plastic pellets and to contribute new insights into the risks associated with plastic pollution at early stages of the plastic life cycle.

## 2 Materials and Methods

### 2.1 Sample Collection

Plastic pellets were collected manually by trained personnel from a single affected beach on the Galician coast (northwestern Spain) following the December 2023 spill (Figure 1). Sampling was conducted along the upper strandline, where sacks of pellets were visibly accumulated. The sampling location corresponded to 42.526540° N, 8.873477° W. Only visually intact pellets were selected to minimize additional contamination.

**Figure 1:**
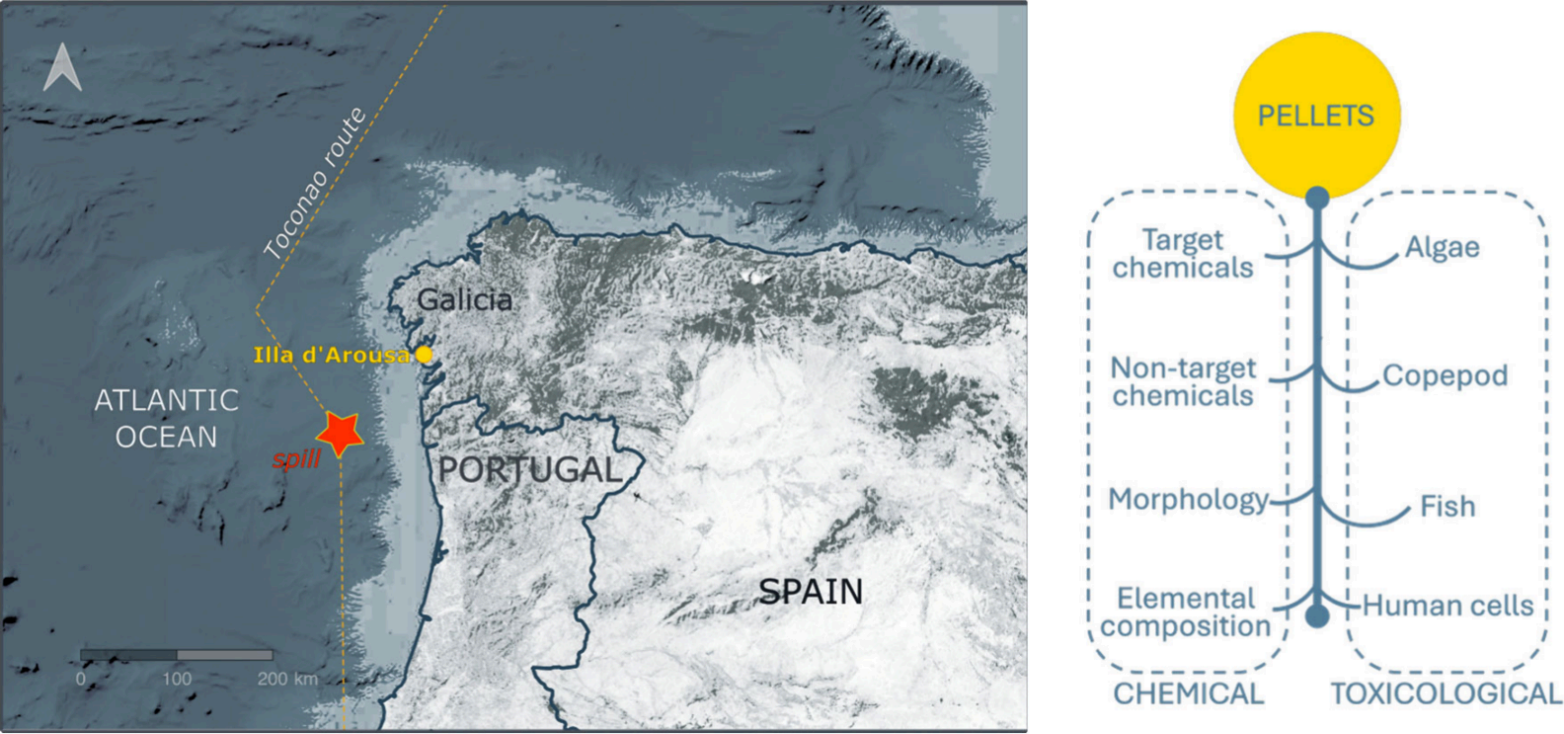
Estimated trajectory and spill site of the Toconao vessel along the Iberian coastline, showing Illa d’Arousa where plastic pellets were collected (left). Overview of the chemical and toxicological analyses conducted on the retrieved pellets, including additive profiling, morphological characterization, determination of the elemental composition, and bioassays with algae, copepods, fish, and primary human cells (right).

After collection, samples were transported to the laboratory, where they were stored in glass containers, kept in the dark at ambient temperature. Chemical and ecotoxicological analyses were initiated within a few days after sampling. Subsamples were distributed to the participating laboratories for chemical and ecotoxicological testing (see SI for details).

### 2.2 Pellet Characterization

The plastic pellets collected from Galician beaches were processed using a range of analytical approaches, including direct analysis of intact pellets without prior extraction, solvent extraction, and leaching protocols, depending on the specific objectives and analytical capabilities of each participating laboratory.

Pellet morphology and elemental composition were assessed using scanning electron microscopy energy-dispersive X-ray spectroscopy (SEM-EDXS) and X-ray fluorescence (XRF) spectroscopy. Chemical characterization of the pellets and a reference sample of Tinuvin UV-622 (Merck & Co., Inc.) was conducted at different laboratories, using a combination of gas chromatography–mass spectrometry (GC–MS), pyrolysis gas chromatography–mass spectrometry (pyGC–MS), and ultrahigh-performance liquid chromatography coupled to tandem mass spectrometry (UHPLC–MS/MS) to target known additives and to screen for the possible presence of other commonly used plastic additives (e.g., benzotriazole UV stabilizers, antioxidants). Nontarget screening (NTS) to assess the presence of other chemicals was conducted using comprehensive two-dimensional gas chromatography–mass spectrometry (GC × GC-MS) for broad chemical profiling. These complementary approaches enabled detailed chemical and elemental characterization of the spilled plastic pellets, identification of additives and NIAS, and evaluation of leachate composition relevant to toxicity testing and environmental risk assessment. A summary of the chemical methods is shown in Table S2 and a detailed description of each method is given in the SI.

### 2.3 Solvent Extracts and Leachate Preparation

To investigate the chemical content of the pellets, solvent extractions were applied to generate extracts for both gas chromatography (GC) and liquid chromatography (LC) analyses, as detailed in the Supporting Information (SI). Aqueous leachates were prepared by incubating pellets in Milli-Q water, artificial seawater (ASW) or sterile water depending on the subsequent chemical analysis and toxicity tests. Leachates were prepared at different pellet-to-water ratios and under slightly different conditions depending on the requirements of each ecotoxicological assay, reflecting differences in test species sensitivity, exposure duration and analytical end points. A detailed description of the aqueous leachate preparation method used for each of the use cases is presented in the Supporting Information (SI). Briefly, leachates were prepared under dark conditions at room temperature (20 °C), using pellet concentrations ranging from 10 to 165 g L^–1^ over time periods ranging from 24 h to 28 days. Reference leachates were also prepared using virgin high-density (HDPE) pellets. For chemical characterization by GC-based techniques, the aqueous leachates were extracted via liquid–liquid extraction (dichloromethane; DCM) after acidification, and the solvent extracts concentrated under nitrogen prior to analysis (see SI for details). For the LC–MS analysis, pellets were subjected to ultrasound-assisted extraction using methanol (MeOH), acetonitrile:methanol (ACN:MeOH, 2:1, v/v), and hexane (Hx). A total of 500 mg of sample was extracted in six cycles (two per solvent, 4 mL each, 2 × 15 min sonication). The combined extracts were evaporated to dryness and reconstituted in MeOH:H_2_O (70:30, v/v).^19^

### 2.4 Toxicity Testing

Toxicity tests were conducted on a range of biological models at different laboratories to assess the effects of plastic pellet leachates An overview of the test species, end points and exposure media are summarized in Table S5.

Models were chosen based on their common use in toxicity testing or for being conservative surrogates, to understanding mechanisms of toxicity of chemicals or in assessing potential to cause environmental impacts. While the selected test species do not represent the local marine biota affected by the Atlantic pellet spill -since two of them are freshwater species (*R. subcapitata* and *D. rerio*) and one (*A. royi*) originates from marine tropical/subtropical regions in Asia -the selected test organisms represent different trophic levels and levels of biological organization (primary producers, invertebrates, vertebrates, and human cells) and are widely used in standardized toxicity testing and chemical risk assessment frameworks (e.g., OECD and ISO guidelines). Their inclusion allows assessment of potential toxic effects across a range of biological systems and exposure scenarios, while ensuring comparability with existing toxicological data sets for plastic-associated chemicals.

#### 2.4.1 Leachate Preparation for Toxicity Texting

Leachates were prepared as described in Section 2.3, using the same general conditions. Minor adjustments in pellet-to-water ratios and exposure durations were applied depending on the requirements of each toxicity test. Detailed protocols for each test system are provided in the SI.

#### 2.4.2 Algal Growth Inhibition Test

The freshwater green microalga *Raphidocelis subcapitata* was exposed to plastic leachates prepared in Milli-Q water. Six exposure treatments were tested, including controls, leachates from Galician pellets prepared at 10 and 30 g L^–1^ after 24 h and 7 d of leaching; and a virgin HDPE reference leachate (30 g L^–1^; 7 d). Algal exposures were conducted in triplicate for 72 h, following OECD guideline (test no. 201). Algal growth inhibition was quantified by chlorophyll-a fluorescence measurements.

#### 2.4.3 Copepod Acute Toxicity Test

The copepod *Apocyclops royi* was exposed to leachates in ASW. Acute toxicity tests followed ISO 14669 guidelines and included an initial range-finding test and a definitive test with multiple concentrations (2–64 g L^–1^). Mortality and sublethal behavioral end points were assessed after 24, 48, and 96 h of exposure. Behavioral end points in *A. royi* included swimming activity and mobility impairment, assessed by visual observation under a stereomicroscope. Tests were conducted in quadruplicate, with five individuals per replicate.

#### 2.4.4 Fish Embryo Toxicity and Locomotion Tests

Fish embryo toxicity tests (FET) were performed on *Danio rerio* embryos less than 4 h postfertilization, following an adapted OECD guideline 236. Embryos were exposed for up to 96 h to control medium, Galician pellet leachates (10 and 30 g L^–1^; 24 h and 7 d), and a virgin high-density polyethylene (HDPE) reference leachate. Each treatment consisted of six replicate wells with seven embryos per well. End points included mortality, hatching success, and morphological abnormalities. Larval locomotion was assessed at 96 h using an automated video-tracking system (ZebraBox, ViewPoint Life Sciences Inc., Montreal, Canada) and analyzed with ViewPoint Zebralab software, which integrates movement activity, distance, and duration across defined time intervals and light/dark cycle.

#### 2.4.5 Human Cell Viability Assay

Primary human peripheral blood mononuclear cells (PBMCs) isolated from healthy donors were exposed to pellet leachates prepared in sterile water. Cells were exposed for 24, 48, and 72 h to increasing leachate volumes corresponding to pellet concentrations of 40 mg L^–1^, 80 mg L^–1^, and 200 mg L^–1^. Cell viability was assessed using the PrestoBlue assay and expressed relative to unexposed controls.

#### 2.4.6 Statistical Analysis

Statistical analyses were performed separately for each test system. For algal growth inhibition, differences among treatments were assessed using one-way analysis of variance (ANOVA) followed by Tukey’s post hoc test for multiple comparisons. For zebrafish embryo mortality, developmental end points and locomotion data, normality was first evaluated using the Shapiro–Wilk test. When assumptions of normality were met, data were analyzed using two-way repeated-measures ANOVA followed by Tukey’s multiple comparisons; otherwise, nonparametric Kruskal–Wallis tests followed by Dunn’s post hoc tests were applied. For PBMC viability assays, donor-level differences relative to controls were calculated and tested against zero using two-sided *t* tests. P-values were adjusted for multiple comparisons using the Benjamini–Hochberg procedure.

All analyses were conducted in GraphPad Prism v10.2.2, GraphPad Software, USA and RStudio 2025.09.1+401. Results were considered statistically significant at *p* < 0.05. Further detail can be found in the SI.

## 3 Results and Discussion

### 3.1 Physicochemical Composition of Pellets

#### 3.1.1 Morphology and Elemental Composition (SEM, EDXS, and XRF)

SEM images (Figure S3). revealed that the spilled pellets were irregular in shape (∼3.25 mm × 2 mm), with one smooth, flat face and a rougher convex side from which filaments projected. Only C and O were detected by EDXS analysis, but these elements cannot be reliably quantified with this technique. With a limit of detection of approximately 1% by weight, any other element, if present, would be at a lower concentration. This assumption was confirmed since the only chemical elements detected by XRF were Ti, Ba and Zn with concentrations of 11.2 ± 1.7 mg kg^–1^, 291 ± 135 mg kg^–1^, and 17.7 ± 4.1 mg kg^–1^, respectively. The pellet density was 1030 ± 10 kg m^–3^ (3σ).

#### 3.1.2 Chemical Composition of Pellets Extracts Compared to Pure Tinuvin UV-622

GC-MS analysis of the pure Tinuvin UV-622 stabilizer revealed the presence of multiple peaks (Figure S4). Although several peaks did not yield a good match to library spectra and could not be identified at an acceptable level of confidence, 5 peaks were identified: phorone (precursor), triacetonamine (UV stabilizer) and a corresponding isomeric structure, and two different methyl esters. Phorone can condense with ammonia to form triacetonamine, confirming a link between these two compounds. Owing to the relatively large molecular size and high boiling point of Tinuvin UV-622, this hindered amine light stabilizer was not detected by GC-MS.

Thermal desorption (TD) of Tinuvin UV-622 produced ∼30 identifiable peaks (Figure S4) including succinic anhydride, methyl esters of butanedioic acid, and triacetonamine, consistent with the results from solvent extracts. Succinic anhydride is most likely a residual chemical from the synthesis of Tinuvin UV-622. Pyrograms of Tinuvin UV-622 (residual material after TD) exhibited a high degree of complexity, as expected for a polymeric structure. Pyrolysis-GC-MS revealed ∼130 compounds that could be tentatively assigned to a structure present in the NIST23 mass spectral library. Many of these were low molecular weight compounds, with the major peaks including succinic anhydride, 3-ethyl-2,4-dimethyl-1H-pyrrole, N-cyano-ethanimidamide, N-(2-propynyl)-N-ethylamine, 2,6-lutidine, as well as a number of hydrocarbons.

Analysis of the pellets resulted in ∼20 peaks being assigned IDs in the TD chromatogram and ∼120 peaks being assigned IDs in the corresponding pyrogram (Figure S4). The pyrogram confirmed the primary polymer to be PE but also contained a distinguishable signal from low-molecular fragments not common to pristine PE. These appeared to derive from the presence of Tinuvin UV-622 (10–13% by mass) in the pellets, with succinic anhydride found in both the TD chromatogram and the pyrogram of the pellets. Other major peaks from the Tinuvin UV-622 pyrograms were also found in the pellet pyrograms. A peak with a retention time of 20.55 min was tentatively identified as Irganox 1076 (Octadecyl 3-(3,5-di*tert*-butyl-4-hydroxyphenyl)­propionate), consistent with the findings from the target GC-MS analysis conducted on the pellets (Section 3.1.3).

Characterization of the pellets and of the pure Tinuvin UV-622 stabilizer reference sample by GC-MS and pyGC-MS confirmed the presence of Tinuvin UV-622 in the spilled pellets collected on the beach. The presence of phorone in both the pure Tinuvin UV-622 and the pellets indicates that this precursor for triacetonamine is not fully reacted and remains in the pellet matrix, or that it may be a degradation product formed in the pellets. Some of the chemicals tentatively identified by TD of the pellets, such as succinic anhydride, may be residuals from the synthesis of Tinuvin UV-622. Differences in the TD chromatographic profiles of the Tinuvin UV-622 and the pellets, for example the tentatively identified thermo-oxidative stabilizer Irganox 1076, suggest the presence of other intentionally added substances or NIAS in the bulk polymer material. The results of the pellet characterization underline the chemical complexity of plastic pellets and provide a clearer understanding of the potential for multiple chemicals to leach from the pellets into the surrounding environment.

#### 3.1.3 Target Analysis of Plastic Pellet Extracts

Target analysis identified 26 known plastic additives (Table S6), including Irganox 1076 (75.2 × 10^3^ ng g^–1^) and degradation products of Irgafos 168 such as 2,4-di*tert*-butylphenol (DTBP) (750 ng g^–1^) and tris­(2,4-di*tert*-butylphenyl) phosphate (TDTBPP) (113 × 10^3^ ng g^–1^) confirming the results from TD-GC-MS analysis (Section 3.1.2). TDTBPP showed the highest concentration among the target analytes. Phthalates, including dibutyl phthalate and dioctyl phthalate, were present in the pellets. Disulfiram was found at high concentrations (7162 ng g^–1^), as well as di­(2-ethylhexyl) tetrabromophthalate (11198 ng g^–1^).

The detected degradation products of the antioxidant Irgafos 168 (DTBP and TDTBPP) are reported to show adverse effects: DTBP is a potential obesogen^20^ and TDTBPP appears to induce reproductive toxicity^21^ and cardiotoxicity^22^ in zebrafish. As additive chemical degradation continues in the marine environment, an increase in the concentrations of DTBP and TDTBPP are expected over time. These additive chemicals and selected degradation products, known for their potential toxic effects, suggest that plastic pellets may act as long-term sources of contamination and highlight the risks of plastic pollution. Furthermore, the presence of degradation products underscores the environmental transformation of these chemicals, potentially increasing their toxicity and persistence.

#### 3.1.4 Target Chemical Analysis of Pellet Leachates

Targeted chemical analysis of ASW leachates revealed a complex mixture of additives spanning multiple functional classes (Table S7), including phthalates, UV stabilizers, antioxidants, flame retardants, antimicrobial agents, bisphenols, parabens and amines. Six phthalates, widely used as plasticizers, were determined in significant concentrations in the leachates. Benzyl butyl phthalate, bis­(2-ethylhexyl) phthalate, and dioctyl phthalate were found at 59.37 μg L^–1^, 224.97 μg L^–1^, and 206.12 μg L^–1^, respectively. Phthalic acid anhydride, a degradation product of many phthalates, was also present at a high concentration (503.26 μg L^–1^). Four UV stabilizers, which protect plastics from degradation by sunlight, were also abundant, including Tinuvin 770 (17 μg L^–1^), Tinuvin RS (5.7 μg L^–1^), and Cyasorb UV-531 (18 μg L^–1^). Three flame retardants present in the leachates included triphenyl phosphate (27.41 μg L^–1^) and tris­(2-chloroethyl) phosphate (8.68 μg L^–1^). Other compounds detected included the antimicrobial agent didecyldimethylammonium chloride (19.03 μg L^–1^) and bisphenol S, a bisphenol A substitute (1.69 μg L^–1^).

#### 3.1.5 Nontarget Analysis of Leachates and Pellet Extracts

Nontarget GC × GC-MS screening of pellet extracts and 7-day leachates identified 24 peaks in MeOH extracts, 55 in MQ leachates, and 51 in ASW leachates (Figure S5). Thirty compounds were common to both aqueous leachates, but the overlap between leachates and MeOH extracts was minimal. Only two compounds, tentatively assigned as phorone and 2,4-dimethylphenol, were present across all matrices. Phorone was the dominant peak in both leachate types but a minor one in the MeOH extract. These results demonstrate that MeOH extraction, although widely used as a proxy for leaching, poorly represents chemical release under aqueous conditions. The comparison of MQ and ASW leachates further indicated that salinity influences both the identity and abundance of leached compounds, consistent with previous studies.^23–25^

While the companies responsible for producing and shipping the spilled pellets stated both the bulk polymer (PE) and the UV-stabilizer (Tinuvin UV-622), most of the chemicals identified by target analysis were not among those reported. NTS revealed additional compounds in the leachates, many of which could only be tentatively identified. Importantly, it is not possible to determine with certainty which of these substances were originally incorporated into the pellets during production (intentionally or NIAS) and which may have been sorbed postspill from the surrounding environment. The lack of transparency regarding the structures and behaviors of chemicals added to plastics during production makes it challenging to identify all of them. As such, the chemical content of individual plastic materials often remains largely unknown to environmental scientists, policy makers and consumers. Furthermore, targeted toxicity testing cannot be conducted on specific ‘unknown’ chemicals, so hazard assessments must rely on more generalized testing of unknown/undefined mixtures, containing unknown substances at unknown concentrations.

Most of the substances identified in the chemical analysis presented in the current study are known plastic additives, some with proven hazardous properties and others where the existing toxicity data is insufficient.^8,9^ These findings highlight how critical the absence of a mandatory and transparent reporting framework for all chemicals incorporated into plastic pellets is, a regulatory gap that undermines efforts to ensure product safety and sustainability. The complex chemical profile of the pellet leachates revealed a range of potentially hazardous additive chemicals and their possible transformation products, pointing to the need for chemical simplification and improvements to existing regulations for reducing environmental impacts. Additionally, there appears to be a degree of redundancy in functionality (e.g., several different plasticizers, UV stabilizers, and flame retardants were identified), which further supports calls for chemical simplification to reduce the complexity of chemicals markets and plastics products.^26,27^ The relatively high concentration of some of the chemicals that are released from the spilled plastic pellets, which can be persistent and harmful, emphasizes the potential ecological risks of plastic pellet pollution. Taken together, the target and nontarget analyses revealed that the spilled pellets contained a diverse and complex mixture of intentionally added substances and NIAS, including synthesis residuals, and transformation products, many with known or suspected hazardous properties.

### 3.2 Toxicity Tests

To assess the potential ecological implications of the chemical complexity described above, we evaluated the adverse effects of pellet leachates using a suite of bioassays.

#### 3.2.1 Effects on Algal Growth

A 72 h exposure of the green microalga *R. subcapitata* to pellet leachates resulted in a significant reduction in algal growth by 18.7 ± 6.3% (*n* = 3) in the 7-d, 30 g L^–1^ treatment relative to media controls (Tukey post hoc test, p-adj = 0.011) (Figure 2). In addition, no significant growth inhibition was observed following 24 h exposure to pellet leachates at 10 g L^–1^ (p-adj = 0.004) and 30 g L^–1^ (p-adj = 0.017). No significant inhibition was detected in the remaining treatments, including those involving virgin HDPE pellets.. These findings are in line with recent studies reporting moderate and time-dependent inhibitory effects of leachates from environmentally exposed pellets, including reduced growth in the diatom *Phaeodactylum tricornutum* and the bacteria *Shewanella* sp., particularly under prolonged exposure.^28^

**Figure 2:**
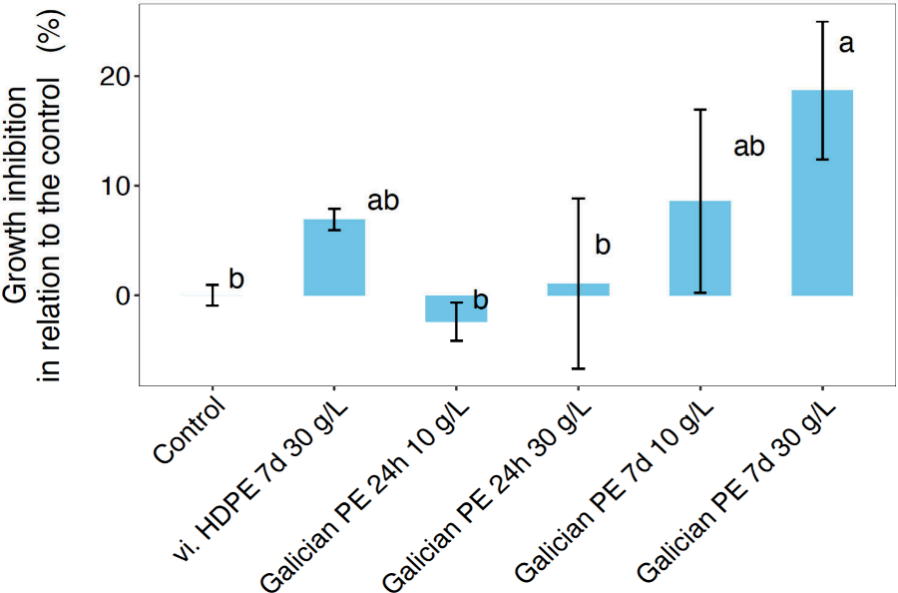
Average algal growth inhibition in relation to the controls (no plastic leachate) when exposed to virgin (“vi.”) HDPE leachate (7 days, 30 g L^–1^) and Galician spill PE pellet for 24 h or 7 days, leachates at 10 or 30 g L^–1^, respectively. Colored bars represent mean values, and error bars represent standard deviation (*n* = 3). The letters on top of the bars show a significant result between treatments obtained using Tukey’s multiple comparisons post-hoc test (*p*-value < 0.05).

Microscopic examination revealed that algae exposed to 7 d, 30 g L^–1^ pellet leachates formed aggregates rather than remaining as solitary planktonic cells (Figure S6). *R. subcapitata* primarily exists as solitary cells, with cell aggregate formation being rare, though under toxic stress it exhibits a multinucleated, palmelloid-like morphology.^29,30^ Other unicellular algae, such as *Chlamydomonas reinhardtii* have shown that cell aggregation is the principal protective mechanism against perchlorate stress.^31^ Cell aggregation has also been reported to be induced by direct contact with microplastic particles.^32^ Microplastic exposure has been shown to elicit stress on algal cells, leading to surface crumpling, extracellular polymer (EPS) secretion, heterogeneous aggregate formation, cell damage, and intracellular material release.^33,34^ The cell aggregation observed points to a certain level of stress in the exposed algae; although the underlying mechanisms remain unclear and the implications of this response warrant further study, the observed aggregation suggests a sublethal stress response that is ecologically relevant given the role of *R. subcapitata* as a primary producer in aquatic food webs.

#### 3.2.2 Effects on Copepod Mortality and Behavior

In range-finding tests, *A. royi* showed no mortality at low leachate concentrations (0.3 g L^–1^, 24 h), while complete mortality occurred at 100 g L^–1^ after 48 h. In the definitive test, mortality remained relatively low (≤25% at 8 g L^–1^ after 96 h) and showed no clear concentration–response pattern, and no significant treatment difference at any of the observed time points (Figure 3, upper panel), suggesting that mortality was not a sensitive end point under the conditions tested.

**Figure 3:**
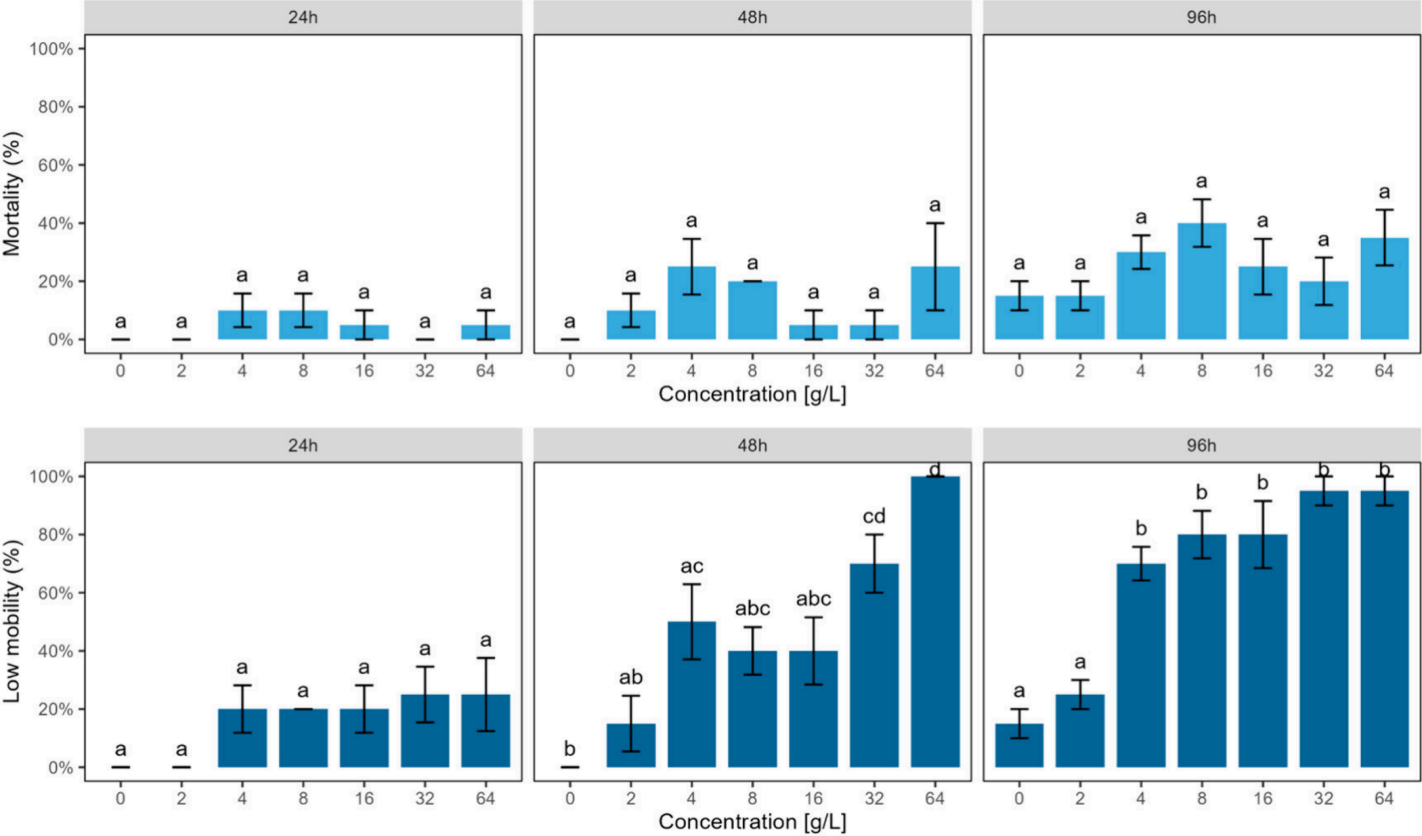
Mortality (upper panel) and behavioral, i.e., low mobility, (lower panel) responses of *Apocyclops royi* after exposure to pellet leachate for 24 h, 48 h, and 96 h, respectively. Behavioral end points showed greater sensitivity than mortality with effects varying by concentration and exposure duration (±SD). Colored bars represent mean values, and error bars represent standard deviation (*n* = 4). The letters on top of the bars show a significant result between treatments obtained using Tukey’s multiple comparisons post-hoc test (*p*-value < 0.05).

In contrast, behavioral end points were considerably more sensitive. Significant (*p* < 0.05) impairments in swimming activity were observed at 32 g L^–1^ and 64 g L^–1^ and after 48 h and starting even at one of the lowest concentrations tested, 4 g L^–1^, after 96 h (Figure 3, lower panel). Impairments in swimming activity were identified, according to Jepsen et al.,^35^ as significant alterations in locomotion, including that swimming only occurred when being touched with a dissection needle. These results indicate that pellet leachates exert time- and concentration-dependent effects on copepod behavior even in the absence of pronounced mortality. Behavioral disruption in copepods is ecologically relevant, as impaired swimming can reduce feeding efficiency and predator avoidance. However, the short exposure durations and relatively high-test concentrations suggest that further work is needed to assess whether such effects occur under environmentally realistic conditions.

#### 3.2.3 Effects on Zebrafish Embryo Development and Behavior

Continuous 96 h exposure of *D. rerio* embryos to pellet leachates and virgin HDPE leachates produced no significant mortality or malformations compared to controls (Table S8). Although hatching success varied slightly among treatments at 72 h, differences were not statistically significant, suggesting that under the tested conditions, zebrafish embryos were less sensitive than algae and copepods.

These findings contrast with the negative effects observed *in R. subcapitata* and *A. royi*, and with previous studies reporting developmental impacts of some chemicals identified in the leachates on *D. rerio* embryos.^36,37^ Such discrepancies may arise from variations in leachate composition (e.g., MQ vs ASW) or from the end points assessed, as the FET test is optimized for acute morphological rather than sublethal or chronic effects, though many of the chemicals associated with plastics can have sublethal impacts, affecting, e.g., metabolic pathways and reproduction.^38^ Furthermore, vessels used to expose zebrafish embryos were of plastic, which may have reduced to bioavailability of the leached chemicals. Overall, the multispecies toxicity approach emphasizes the need for integrated assessments across taxa and exposure conditions. Expanding the analysis to longer exposures, mixed toxicity, and sublethal end points could provide a more comprehensive understanding of the ecological hazards posed by these pellets.

#### 3.2.4 Effects on Viability of Human Peripheral Blood Mononuclear Cells (PBMCs)

PBMC viability was not significantly affected after 24 h exposure to pellet leachates, regardless of pellet number (10, 20, or 50 pellets) or leachate volume (10, 30, or 50 μL) (Figure 4). Leachates were generated using defined pellet loads (10, 20, or 50 pellets per extraction volume), corresponding to nominal concentrations of 40, 80, and 200 mg L^–1^, respectively, which are of the same order of magnitude as those applied in the other toxicity assays (see Section 2.4).

**Figure 4:**
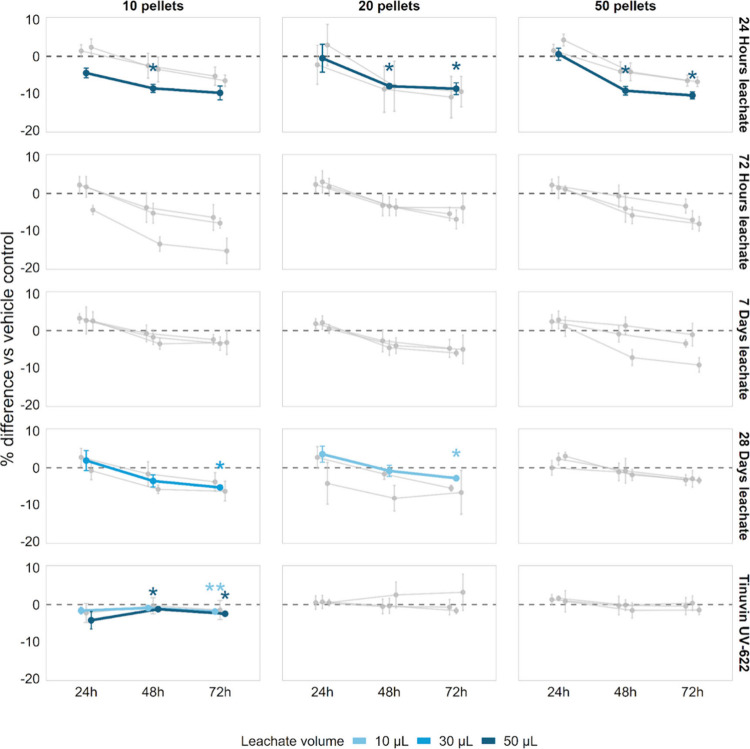
Effects of plastic pellet leachates on the viability of human PBMCs. Changes in PBMC viability across exposure conditions are shown as the mean percent effect difference relative to the vehicle control (±SD) from the three donors over time. Series with at least one significant change (adjusted *p* < 0.05) at any time point are highlighted by colors according to the leachate volume.

After 48 h, a consistent trend toward reduced cell viability was observed across exposure conditions, with the strongest effects occurring at the highest applied leachate volume (50 μL). Specifically, PBMC viability decreased by 8.7% (*p* = 0.05) for leachates generated from 10 pellets, by 8.1% (*p* = 0.02) and 8.8% (*p* = 0.05) for 20 pellets after 48 and 72 h, respectively, and by 9.3% (*p* = 0.02) and 10.6% (*p* = 0.02) for 50 pellets after 48 and 72 h, respectively.

Leachates generated from longer dissolution times (72 h, 1 week, or 1 month) did not produce consistent effects across exposure durations, although isolated statistically significant reductions in viability were observed (e.g., 28-day leachate: 10 pellets, 72 h exposure, 30 μL, 5.3% decrease, *p* = 0.02; 20 pellets, 72 h exposure, 10 μL, 2.8% decrease, *p* = 0.02). Exposure to the reference plastic additive Tinuvin UV-622 resulted in minor changes in PBMC viability, ranging from a 4.2% decrease to a 3.3% increase.

Overall, pellet leachates induced modest, time-dependent reductions in PBMC viability, with maximum effects below 15% relative to controls. While acute exposure caused minimal effects, delayed responses observed after prolonged exposure suggest the potential for cumulative or time-dependent impacts on immune cell integrity.

Although the observed reductions in PBMC viability were modest, their consistent appearance after prolonged exposure suggests a time-dependent cellular response rather than acute cytotoxicity. Similar patterns of limited short-term effects combined with delayed or cumulative responses have been reported for human cells exposed to plastic-associated chemical mixtures, supporting the interpretation that pellet leachates may interfere with cellular processes without causing rapid cell death.^39,40^ In this context, PBMCs provide a relevant human immune cell model to detect subtle, sublethal effects that may not be apparent in acute exposure scenarios. Differences among leachates generated with varying dissolution times likely reflect changes in the composition and availability of leachable additives over time, highlighting the importance of exposure duration when assessing potential human health hazards associated with plastic pellets.

### 3.3 Broader Environmental and Policy Implications

The results from the study collectively reveal that the *Toconao* pellet spill in Galicia represents a complex and chemically active pollution event with implications that extend beyond the immediate visual impact of plastic contamination. The chemical characterization of the pellets demonstrated a high diversity of both intentionally and nonintentionally added substances, including several compounds with known or suspected hazardous properties beyond those reportedly present in the pellets (Tinuvin UV-622). This complexity, coupled with the incomplete information provided by the manufacturer, highlights persistent gaps in transparency regarding the composition and potential risks of preproduction plastics. Toxicological assays confirmed that the pellets are not chemically inert materials but active sources of chemicals liberated into aqueous leachates capable of inducing measurable biological effects in model organisms from different trophic levels. The combination of chemical and biological evidence underscores that the environmental hazard of spilled pellets is driven not only by their physical presence as microplastics, but also by their chemical burden and the potential for additive and degradation products to exert sublethal or cumulative effects over time, especially linked to the high environmental pellet concentrations that occur at hot spots or in spill events.

Overall, three key observations emerge from this study: (a) pellet spills constitute a recurrent and chemically complex source of pollution, whose impacts can persist well beyond the initial cleanup phase; (b) current product information and regulatory frameworks remain insufficient to capture the full spectrum of chemical hazards associated with preproduction plastics; (c) observed biological effects emphasize the need for precautionary, upstream measures to prevent losses and minimize the environmental footprint of pellet handling and transport.

Together, these findings integrate scientific evidence and frame the broader implications of the *Toconao* pellet spill, emphasizing persistent regulatory gaps and the need for stronger prevention and response strategies. While previous incidents have prompted cleanup responses,^41–43^ conventional cleanup methods, such as fine mesh nets and vacuum systems, are often insufficient to fully address the scope and complexity of pellet pollution.^44^ Many pellets remain buried in sediments or dispersed by ocean currents, weathering and liberating chemicals to their ambient environment over time, complicating removal efforts and risking further ecological disturbance, such as the damage of benthic habitats or inadvertent burial of pellets. These limitations highlight that prevention, rather than remediation, must be the primary focus of management frameworks.

From a scientific perspective, the toxicological assays applied in this study provide complementary lines of evidence on the potential impacts of pellet-derived chemical mixtures across biological levels, rather than aiming to define environmentally representative exposure thresholds. The selected test organisms are widely used models in ecotoxicology and toxicology, allowing the detection of mechanistic and sublethal responses to complex chemical mixtures, including growth inhibition, behavioral alteration, developmental effects, and reduced cell viability. Although the tested concentrations exceed typical background environmental levels, they reflect realistic conditions at contamination hot spots and spill scenarios, where pellets can accumulate at very high densities. Differences in exposure media, temperature, and test design across assays are acknowledged as a limitation for direct cross-species comparison; however, they also reflect the diversity of environmental compartments and biological sensitivities potentially affected by pellet spills. Together, these results highlight that pellet-derived chemicals can induce biologically relevant effects under plausible spill-related exposure conditions, reinforcing the need to address pellets not only as physical pollutants but also as chemically active stressors.

The *Toconao* spill also underscores the importance of citizen-led monitoring and rapid response, as documented by Vidal-Abad et al.,^45^ while also highlighting the need for stronger international coordination to address transboundary pellet pollution. Historically, European legislative frameworks have lacked enforceable provisions to prevent pellet loss at sea. In response, the EU Council adopted specific requirements in December 2024 for the transport of plastic pellets by sea, mandating improved packaging standards and cargo documentation.^46^ Although implementation was initially expected to be delayed to align with future actions by the International Maritime Organization (IMO), by September 2025 the EU had already adopted the first regulation addressing pellet losses across the entire supply chain, introducing binding obligations, certification schemes, risk-management plans, and annual loss reporting.^47^

At the global level, the IMO’s MARPOL Convention provides overarching guidelines on ship-source pollution but does not currently classify plastic pellets as hazardous cargo. Recent progress within the IMO nevertheless signals growing momentum. Amendments adopted by the Marine Environment Protection Committee (MEPC) in March 2024 strengthened pellet transport safety measures, including container securing and stowage requirements,^48^ followed by best-practice guidelines for pellet spill response approved at MEPC 82.^49^ In 2025, the Pollution Prevention and Response Sub-Committee (PPR 12) finalized a revised Action Plan to Address Marine Plastic Litter from Ships, endorsed at MEPC 83, which includes the development of mandatory measures to reduce environmental risks associated with pellet transport.^50^

Although completely eliminating pellet spills at sea may only be achievable if maritime transport of pellets is phased out, several measures can already reduce both the likelihood and the impact of spills. A critical step is to classify plastic pellets as dangerous goods under the International Maritime Dangerous Goods (IMDG) Code,^51^ strengthening requirements for packaging, handling, liability, and compensation. Such a classification would be firmly grounded in scientific evidence on pellet composition, environmental fate, and toxicity, and would align pellet transport with other hazardous cargoes. Comparable national precedents illustrate the feasibility of this approach: for example, Canada classified plastic microbeads as harmful substances under Schedule 1 of the Canadian Environmental Protection Act (CEPA), a framework later used to ban several categories of single-use plastics.^52,53^ These experiences also highlight the political and legal challenges associated with regulating plastic products under existing toxic substances legislation.

Frequent pellet spills across regions underscore that pellet pollution is a persistent and transboundary challenge. The new EU Regulation marks a turning point by introducing enforceable measures that strengthen accountability and prevention throughout the entire supply chain, setting an important precedent for other jurisdictions. Yet, the global dimension of pellet transport and trade demands broader coordination. While recent progress within the IMO toward harmonized maritime safety standards is encouraging, sustained international commitment will be essential to close remaining regulatory gaps and effectively reduce the long-term risks and recurrence of pellet pollution.

## Supplementary Material



## Notes

Overview of reported shipping-related pellet spill events (Table S1), detailed methodological descriptions for chemical characterization and leachate preparation, supplementary analytical tables (Tables S2–S8), additional figures (Figures S1–S6), toxicity test protocols, and extended statistical analyses supporting the results presented in the main manuscript (PDF)

## ◆

J.B.: Deceased on September 27, 2024.
