# Supplementary material for: Integrated Chemical and Hazard Assessment of Plastic Pellets from the Toconao Spill (Galicia, Spain) Indicates Potential for Environmental Harm
Source: Environ Sci Technol. 2026 Feb 19;60(8):6696–706. doi: 10.1021/acs.est.5c16166 (PMC12961947; doi:10.1021/acs.est.5c16166)
Supplement: Supplementary file 1 [file es5c16166_si_001.pdf]

Integrated Chemical and Hazard Assessment of Plastic Pellets from the *Toconao* Spill (Galicia, Spain) Indicates Potential for Environmental Harm

Carmen Morales-Caselles[1,2]*, Andy M. Booth[3], Juan Baztan[4]◆, Line-Marie Berget[5], Eric Carmona[6,7], Natàlia Corcoll[8], Hubert Dirven[5], Montserrat Filella[9], Daniela Gómez-Martínez[8], Dorte Herzke[10,11], Hege Hjertholm[5], Annika Jahnke[6,12], Per Meyer Jepsen[13], Azora König Kardgar[8], Claudia Lorenz[13], Neema Negi[5], Elisa Rojo-Nieto[6], Igor Snapkow[5], Lisbet Sørensen[3,14], Kristian Syberg[13], Hideshige Takada[15], Andrew Turner[16], Bethanie Carney-Almroth[8]*

[1] Biology Department, Faculty of Marine and Environmental Sciences, University of Cádiz, Puerto Real, Cádiz, 11510, Spain
[2] Institute of Marine Research (INMAR), University of Cádiz, Puerto Real, Cádiz, 11510, Spain
[3] SINTEF Ocean AS, Department for Climate and Environment, NO-7465 Trondheim, Norway
[4] Versailles Saint-Quentin-en-Yvelines University, 78035 Versailles, France
[5] Department of Chemical Toxicology, Norwegian Institute of Public Health, NO-0213 Oslo, Norway
[6] Department of Exposure Science, Helmholtz Center for Environmental Research (UFZ), 04318 Leipzig, Germany
[7] Department of Atmospheric Chemistry, Leibniz Institute for Tropospheric Research (TROPOS), 04318 Leipzig, Germany
[8] Department of Biological and Environmental Sciences, Centre for Future Chemical Risk Assessment and Management Strategies (FRAM), University of Gothenburg, 40530 Gothenburg, Sweden
[9] Department F.-A. Forel, University of Geneva, 1205 Geneva, Switzerland
[10] Division of Climate and Environmental Health, Norwegian Institute of Public Health, NO-0213 Oslo, Norway
[11] NILU, NO-9296 Tromsø, Norway
[12] Institute for Environmental Research, RWTH Aachen University, 52074 Aachen, Germany
[13] Department of Science and Environment, Roskilde University, 4000 Roskilde, Denmark
[14] Norwegian University of Science and Technology (NTNU), Department of Chemistry, NO-7491 Trondheim, Norway
[15] Laboratory of Organic Geochemistry, Tokyo University of Agriculture and Technology, 183-8509 Tokyo, Japan
[16] School of Geography, Earth and Environmental Sciences, Plymouth University, PL4 8AA Plymouth, United Kingdom

*Corresponding authors: carmen.morales@uca.es ; bethanie.carney@bioenv.gu.se

◆ *J.B. Deceased on September 26, 2024.*

Summary information: Number of Pages: 23; Paragraphs: 1; Tables: 8; Figures: 6

Table S1. Overview of known or reported shipping-related pellet spills since 2010.

**Date**	**Location**	**Vessel**	**Incident Description**	**Number of Nurdle Containers Lost**	**Source**
2025	India	MSC ELSA 3	Capsized	~71,500 sacks on board; ~858 sacks (~22 t) recovered	Perumal et al (2025)
2025	North Sea	Solong container ship	collision with MV Stena Immaculate tanker	~15 containers lost; ~10 t recovered onshore	News
2023	Portugal/Spai n	TOCONAO	Container loss	6	News
2023	Türkiye	Containers not vessels	Fire due to an earthquake in Iskenderun Port	Unknown	News
2021	Sri Lanka	M.V. X-PRESS PEARL	Fire and sinking	Unknown	Sewwandi et al (2023)
2020	New Orleans, USA	CMA CGM BIANCA	Container loss	4	Simard et al (2024)
2020	South Africa	Undisclosed	Container loss	6	-
2020	North Sea	TRANS CARRIER	No containers lost; nurdles directly spilled into sea	0 (13.2 MT released)	Pew Charitable Trust
2019	Netherlands	MSC ZOE	storm	1 container with nurdles (342 in total) 22.5 tonnes	Le Cedre
2017	Durban, South Africa	MSC SUSANNA	Container spill	2	www.itopf.org
2012	Hong Kong	Unknown	Typhoon	5	www.itopf.org
2011	New Zealand	M.V. RENA	Grounding	4	www.itopf.org

Paragraph S1. Detailed Methods

Solvent-based extraction of pellets

For chemical analysis of the pellets by LC-based techniques, extraction was performed using a solvent mixture of *n*-hexane and dichloromethane (50:50, v/v). Five pellets (approximately 0.1 g) were placed in a 30 mL amber glass vial and 30 mL of the solvent mixture was added. The extraction was conducted in the dark for 24 hours, after which the solvent was removed and replaced with fresh solvent. This process was repeated three times for a total extraction time of 72 hours. The combined extracts were evaporated with nitrogen and reconstituted in 250 µL of methanol (MeOH) for further analysis. For GC×GC-MS analysis, pellets were extracted by MeOH (five pellets to 4 mL) at room temperature in an ultrasonication bath (60 mins). The extract was evaporated under nitrogen to ~0.6 mL for analysis.

Leachate preparation

For LC-MS and GC×GC-MS characterization, leachates were prepared in a ratio of 30 g/L in both MilliQ (MQ) and artificial seawater (prepared as described above) for 7 days at room temperature (20°C) in the dark. Procedural blanks of each water type were included. For the GC×GC-MS analysis, the pellets were removed at the end of the incubation period, the water was acidified (HCl, pH <2), and then extracted by liquid-liquid extraction using (dichloromethane (DCM). The aqueous leachates were extracted three times using 30 mL of DCM, the extracts were then combined and dried using anhydrous sodium sulphate. Finally, the extracts were then concentrated to ~0.5 mL under nitrogen before analysis. For LC-MS analysis,leachates were extracted with a SPE method using Oasis HLB cartridges (Waters, Milford, MA, USA). Cartridges were conditioned sequentially with 5 mL ethyl acetate (EtAc), 5 mL methanol (MeOH), and 10 mL LC–MS grade water. Leachate loading was performed at a flow rate of approximately 10 mL min^{-1}. After application, cartridges were rinsed with 5 mL LC–MS grade water and dried under vacuum for 30 s. Cartridges were subsequently dried under a high-purity nitrogen stream for 1.5 h. Elution was carried out consecutively into the same collection vial with 2.5 mL EtAc, 2.5 mL MeOH, 2.0 mL MeOH containing 1% formic acid, and 2.0 mL MeOH containing 2% (v/v) 7 N ammonia in MeOH.

For toxicity testing, leachates were prepared for use in different model systems and were therefore prepared in a number of different ways. For zebrafish and microalgal exposures, the PE pellets from the Galician spill accident were leached in Milli-Q water, at room temperature (20°C) and in the dark (shielded from light with aluminum foil wrapping). Two leaching periods of 24 hours and 7 days and two concentrations of plastic pellets of 10 g/L and 30 g/L were applied. Additionally, 30 g/L virgin HDPE pellets were leached for 7d using the same protocol as the Galician pellets (GP) as a reference. For human cell toxicity testing Plastic pellets were also leached in sterile water (Fresenius, Germany). 5 mL solutions were prepared with 0, 1, 5, 10, 20 and 50 pellets (0, 3.3, 16.5, 33, 66, 165 g/L) with leaching periods of 24 hr, 72 hr, 7 d and 28 d. All leaching was done in the dark at room temperature after 1 hour of ultrasonication. After the leaching process, the supernatant was collected and stored at 4 degrees until further analysis. Plastic Pellet additive 11 (Merck & Co., Inc.) was used as a standard for experiments.

Leachates were also prepared with artificial seawater, created by dissolving 33 g/L of Instant Ocean® Sea Salt in Milli-Q water. This mixtures contains 380-400 mg/L calcium, 1180-1120 mg/L magnesium, 359 mg/L potassium, 8.5-9.5 °dKH, 222 mg/L hydrogen carbonates, 7190 ug/L strontium, <0.2 mg/L zinc, 76 mg/L bromides, <5 mg/L fluorides, <10 mg/L iodides, 18300 mg/L chlorides, 9850 mg/L sodium, 1498 mg(SO4)/L sulfates, <4 ug/L antimony, 5800 ug/L boron, 369 ug/L barium, <4 ug/L cobalt, 23 ug/L lithium, <4 ug/L molybdenum, <4 ug/L selenium, and <4 ug/L vanadium. For copepod exposure leachates were prepared at 100 g/L in artificial seawater (prepared as described above), where the PE pellets were incubated at room temperature (20°C) in the dark for 7 days.

CHEMICAL ANALYSIS

Table S2 Overview of chemical analysis methods applied to plastic pellet samples, including target and non-target chemicals analyzed and sample media. SEM-EDXS: Scanning Electron Microscopy coupled with Energy-Dispersive X-ray Spectroscopy; XRF: X-ray Fluorescence; GC–MS: Gas Chromatography–Mass Spectrometry; pyGC–MS: Pyrolysis Gas Chromatography–Mass Spectrometry; UHPLC–MS/MS: Ultra-High-Performance Liquid Chromatography coupled with Tandem Mass Spectrometry; GC×GC–MS: Comprehensive Two-Dimensional Gas Chromatography–Mass Spectrometry; NTS: Non-target screening.

Analytical method	Chemicals analyzed	Medium
SEM-EDXS	Imaging, elemental analysis	None
XRF	As, Ba, Bi, Br, Cd, Cl, Cr, Cu, Fe, Hg, Ni, Pb, Sb, Se, Sn, Ti, V, Zn	None
GC-MS	Tinuvin UV-622 (reference chemical), benzotriazole UV stabilizers, antioxidants (Irganox1076 and Irgafos168, including degradation products)	None (UV-622), solvent extract
Thermal desorption and pyGC-MS	Tinuvin UV-622 (reference chemical), polyethylene, NTS	None
UHPLC-MS/MS	Target analysis of plastic-associated chemicals	Solvent extract, leachate
GC×GC-MS	NTS	Solvent extract, leachate

Scanning Electron Microscopy (SEM)- Energy-dispersive X-ray Spectroscopy (EDXS)

A pellet was mounted on an aluminium stub with double-sided conductive carbon tape and then coated with gold (ca. 10 nm) using low vacuum sputter coating. A JEOL JSM-7001F scanning electron microscope (Department of Earth Sciences, University of Geneva, Switzerland), equipped with an EDXS detector (model EX- 94300S4L1Q; JEOL) was used to obtain images of

the pellet and perform EDXS analyses. EDXS measurements were acquired with settings of 15 kV accelerating voltage, a beam current of 7 nA and acquisition times of 30 s.The whole energy range (0–20 keV) was acquired and used for element detection. EDXS results are presented as mol %, with detection limits dependent on several parameters but estimated to be around 1%.

X-ray fluorescence (XRF) analysis

Various elements (As, Ba, Bi, Br, Cd, Cl, Cr, Cu, Fe, Hg, Ni, Pb, Sb, Se, Sn, Ti, V, Zn) were analysed in the pellets by energy-dispersive X-ray fluorescence (XRF) spectrometry using a Niton XL3t 950 He GOLDD+ configured in a laboratory test stand. The instrument was operated in a standardless ‘plastics’ mode and with a 3-mm thickness correction applied. Pellets were placed, individually, above the detector window and irradiated with an 8-mm diameter beam, with counting performed for 120 s in total (60 s each in low and main energy modes). Resulting spectra were quantified by fundamental parameter coefficients to yield concentrations on a dry weight basis (in mg kg^{-1}) and with a counting error of 2s (95% confidence). As a performance check, a polyethylene reference disc (Niton PN 180-619) was analysed at the beginning and end of the pellet measurements, with the instrument returning concentrations that were within 15% of certified values. Detection limits varied depending on the precise pellet characteristics but in general ranged from < 4 mg kg^{-1} for As, Br, Pb and Se to about 25 mg kg^{-1} for Cd, Cl and Se; however, because the energy of the main fluorescent X-rays for Ba were towards the maximum irradiation energy, the detection limit for this element was around 150 mg kg^{-1}.

Pellet density analysis

The density of the pellets was measured at room temperature (20 + 0.1 °C) with a Quantachrome Ultrapyc 1200e multi-volume gas pycnometer (Department of Earth Sciences, University of Geneva, Switzerland). Around 1 g of sample was weighed to three decimal places to fill a stainless-steel chamber that was then sealed. Helium from an ultrahigh purity compressed source was introduced to the chamber to purge and condition the sample before the pressure was left to stabilise and recorded. The valve of the pycnometer was opened and a few minutes later the reduced pressure was measured. For replication, the process was repeated five times which provided an acceptable relative standard deviation. Density was subsequently computed from the mean displacement volume and sample mass.

Analysis of Tinuvin UV-622 by GC-MS

Tinuvin UV-622 was dissolved in dichloromethane (2 mg/mL) and analyzed using an Agilent 7890A GC coupled to a 5975C MS. 1 µL was injected in pulsed splitless mode at 310 C and separation achieved using a HP-5 (30m x 0.25 mm x 0.25 µm) column with He (1.1 mL/min) as mobile phase. The column oven was programmed from 60 °C (2 min hold), ramped by 15 °C/min until 325 °C and held for 10 minutes. The MSD transfer line was held at 325 °C. The solvent delay was 6 minutes. The MS was operated in fullscan mode (mass range 50-550 m/z) at 70 eV ionization, with ion source and quadrupole temperatures 230 and 150 °C, respectively.

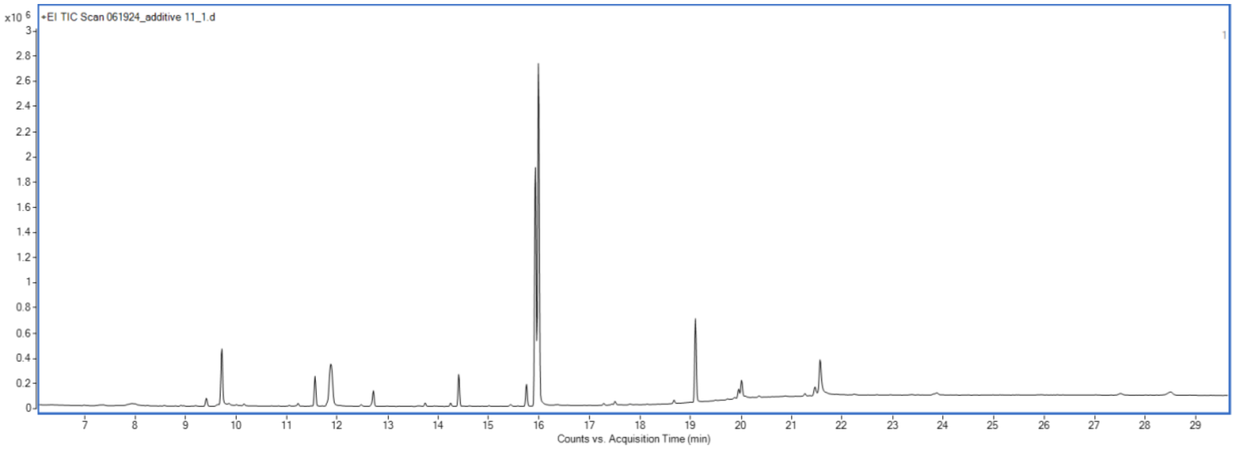

Figure S1. GC-MS chromatogram of pure UV622 dissolved in DCM.

Thermal desorption and pyrolysis GC-MS analysis

Subsamples of the pellets (~0.25 mg cut by scalpel from the pellet core) and the UV622 reference material were subject to thermal desorption and pyrolysis GC-MS analysis. Samples were weighed in stainless steel cups and covered with glass wool. Analysis was achieved using a Frontier Mulit-Shot Pyrolyzer (PY-3030D) coupled to an Agilent 7890A GC with an Agilent 5975C MS. The pyrolyzer was operated in double-shot mode, with initial thermal desorption of the sample at 150-300 °C (initial time 0.10 min, 100 °C/min rate, hold 1 min), followed by pyrolysis at 600 °C (1 min). The interface and inlet temperatures were 320 °C. The split ratios were 25:1 and 100:1 for thermal desorption and pyrolysis analysis, respectively. The carrier gas was helium at a constant flow of 1 mL/min. Separation was achieved using a HP5-MS UI capillary column (30 m length, 0.25 µm film thickness and 0.25 mm internal diameter). The column oven temperature was programmed at 40 °C (2 min), ramped by 20 °C/min until 320 °C (25 min hold). The transfer line temperature was 320 °C, the ion source temperature 230 °C and the quadrupole temperature 150 °C. The ion source was operated in fullscan mode (29-600 *mz*) at 70 eV.

Target analysis of organic additives

Instrumental analysis was performed on a UHPLC-MS/MS system (Agilent 1290 Infinity coupled to an Agilent 6490 triple quadrupole LC/MS, Agilent Technologies, Santa Clara, USA) with an ESI source. Chromatographic separation was performed on a Zorbax Eclipse Plus C18 column (95 Å, 2.1 mm × 150 mm, 1.8 µm, Agilent Technologies, Santa Clara, USA). Multiple reaction monitoring (MRM) was used with mass spectrometric parameters optimised for each analyte. Quantification was performed using a 14-point external calibration curve (0 - 250 ng mL-1). Samples were analysed using the method developed at the UFZ and published at Menger et al. 2024 for plastic-associated chemicals (n = 71), including plasticisers (n = 13), UV stabilisers (n = 21), antioxidants (n = 11), flame retardants (n = 17), precursor monomers (n = 8) and others (n = 1).

Additional target analysis was performed on the extracts and leachates of beached pellets using an Orbitrap Exploris 120 mass-spectrometer equipped with Vanquish UPLC system (Thermo

Scientific). The column was ACQUITY UPLC HSS T31.9 mm, 2.1x100 mm (Waters, p/n 186003539). Both positive and negative ions were monitored in the m/z range of 200-3000. Mass resolution was set at 60000 for full-scan and at 15000 for data-dependent MS-MS experiments. For the LC method, Eluent A was 2 mM ammonium acetate in 10% methanol in water, Eluent B was 2 mM ammonium acetate in methanol, and the elution rate was 0.35 mL/min. The gradient was as shown on Figure S2.

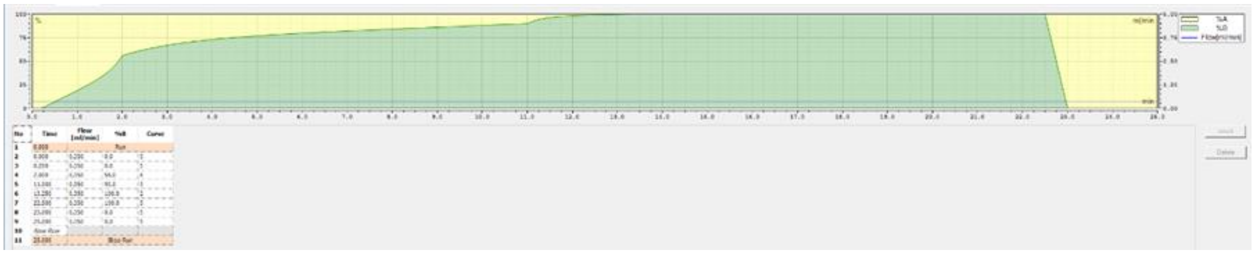

Figure S2. Solvent gradient for UPLC-HRMS method

Target analytical procedure for benzotriazole UV stabilizers (BUVSs)

Five pellets (~ 0.1 g) were weighed and put in a 30-mL glass amber vial, ~15 mL of hexane was added and the vial was kept in the dark at room temperature for 24 h. The hexane solution was then transferred with glass pipette to pear-shape flask; fresh solvent (hexane) added and the extraction repeated. These sequential extracts obtained through the 72-h-extraction were combined, concentrated by rotary evaporator, re-dissolved into ~ 0.5 mL of hexane, and subjected to purification for instrumental analysis, which was described in Matsunaga et al. (2025). Operation conditions of GC-MS for BUVSs are listed in Table S2 and monitored ions on GC-MS are listed in Table S3.

Target analytical procedure for Antioxidants (AOs: Irganox1076 and degradation products of Irgafos168).

Twenty five pellets (~ 0.1 g) were extracted with hexane as described above. The extracts were combined to be ~ 50 mL. Approximately 2 mL aliquot was taken in a 2 mL glass ampoule and solvent was evaporated to dryness under gentle stream of nitrogen and filled-up with 100 µL of isooctane containing chrysene-d_{12} as injection internal standard. One µL aliquots were injected to GC-MS for quantification of Irganox1076 and degradation products of Irgafos168 (2,4-di-tert-butylphenol (2,4-DTBP) and tris (2,4-di-tert-butylphenyl) phosphate (TDTBPP). Operation conditions of GC-MS for AOs are listed in Table S3 and monitored ions on GC-MS are listed in Table S2.

Non-target screening of organic chemicals in pellets and leachates

Comprehensive two-dimensional gas chromatography-mass spectrometry (GC×GC-MS) analyses were performed using a 7890B GC coupled with a 7250 quadrupole time of flight mass spectrometer interfaced with a Zoex ZX2 cryogenic modulator. The first-dimension column was a Zebron ZB-1plus (30 m × 0.25 mm × 0.25 µm) and the second-dimension column was a BPX50 (1.0 m × 0.25 mm × 0.25 µm), interfaced by a 1 m × 0.25 mm deactivated fused silica modulation loop. The carrier gas was high purity helium at constant flow (1.1 mL/min). Samples (1 µL) were injected at 250 °C splitless. The oven temperature was kept at 60 °C (1 min hold), ramped by 5 °C/min to 300 °C (10 min hold). The hot jet was offset at +10 °C (1 min hold), ramped by 7 °C/min to 360 °C (10 min hold). The hot jet was. The modulation time was 6 s with a 350 ms pulse length. The transfer line temperature was 300 °C, the ion source temperature was 200 °C, the quadrupole temperature 150 °C. The EI source was operated at 70 eV. Scan speed was 50 Hz, and the recorded mass range 50-650 m/z. A standard mixture was co-injected at least every ten samples. MassHunter Unknowns Analysis software was applied to the raw data files for deconvolution and tentative identification of analytes using a query towards NIST25.L, returning the best matches (up to five for each peak) with a similarity of > 80 %. Output files with all peaks, as well as with assigned tentative identities were then exported to .csv format for further processing using R[1]. First dimension retention indexes (RIs) were calculated for all tentatively identified compounds [2]. For GCxGC-MS, comparing first dimension RIs and second dimension absolute retention times predicted from physical-chemical properties (automatically retrieved from PubChem using pubchempy) were used to filter off unlikely matches using partial least squares prediction[3,4], leaving only tentatively identified compounds with less than 300 deviation from predicted RI and less than 2 s absolute deviation of second dimension retention time. Manual inspection of results was applied to combine peaks with multiple assignments. Limits of detection are calculated as the average of (at least 3) blanks plus 3 times standard deviation. Only results above the limit of detection are taken further. If internal standards are added, it is good practice to normalize responses to those prior to LOD assignment and any further data analysis.

A sample of Tinuvin UV622 plastic additive, as well as extract and leachate samples of the beached pellets were analyzed on Orbitrap Exploris 120 mass-spectrometer equipped with Vanquish UPLC system (Thermo Scientific). The column was ACQUITY UPLC HSS T31.9 mm, 2.1x100mm (Waters, p/n 186003539). Both positive and negative ions were monitored in the m/z range of 200-3000. Mass resolution was set at 60000 for full-scan and at 15000 for data-dependent MS-MS experiments. The target compound names, m/z ratios and retention windows for target screening in negative mode are listed in Table S2.

Table S3. Target list for LC chemical analysis in negative mode

Compound	*m/z*	T start (min)	t stop (min)
4:2 FTS	326.9743	5.5	15
6:2 FTSA	426.9679	5.5	15
8:2 FTSA	526.9615	5.5	15
EtFOSA	525.9775	5.5	15
NEt-FOSAA	583.983	5.5	15

EtFOSE	630.0249	5.5	15
7:3 FTCA	440.9977	5.5	15
FOSA	497.9462	5.5	15
FOSAA	555.9517	5.5	15
5:3 FTCA	341.0041	5.5	15
3:3 FTCA	241.0105	5.5	15
HFPO-DA	328.9677	5.5	15
MeFOSA	511.9619	5.5	15
NMe-FOSAA	569.9673	5.5	15
MeFOSE	616.0092	5.5	15
NaDONA	376.9689	5.5	15
PFBA	212.9792	3	15
PFBS	298.943	3	15
PFDA	512.96	5.5	15
PFDoDA	612.9537	5.5	15
PFDiS	598.9238	5.5	15
PFHpA	362.9696	5.5	15
PFHpS	448.9334	5.5	15
PFHxA	312.9728	5.5	15
PFHxDA	812.9409	5.5	15
PFHxS	398.9366	5.5	15
PFNA	462.9632	5.5	15
PFNS	548.927	5.5	15
PFOA	412.9664	5.5	15
PFOcDA	912.9345	5.5	15
PFOS	498.9302	5.5	15
PFPeA	262.976	4	15
PFPeDA	762.9441	5.5	15
PFPeS	348.9398	5.5	15
PFTeDA	712.9473	5.5	15
PFTrDA	662.9505	5.5	15
PFUnDA	562.9568	5.5	15
PFUnDS	648.9206	5.5	15

Table S4 Compounds used for training of the GCxGC PLSR model

**Compound class**	**Compound Name**	**Short name**	**CAS**	**Rt 1D (min)**	**Rt 2D (s)**
Hydrogenated PAH	Decalin, cis		91-17-8	9.93	0.96

Phenol	2,4-Dimethylphenol		105-67-9	10.88	1.85
Phenol	4-Ethylphenol		123-07-9	11.31	1.92
Phenol	3,5-Dimethylphenol		108-68-9	11.41	1.86
Hydrogenated PAH	Methyldecalin		2958-76-1	11.79	0.99
Aromatic	Benzothiophene		270-82-6	11.86	2.58
Phenol	2,4,6-Trimethylphenol		527-60-6	12.26	1.93
Plastic/rubber chemical	Benzothiazole		95-16-9	12.56	3.26
n-alkanes		nC12	112-40-3	12.91	0.48
Plastic/rubber chemical	n-Cyclohexylformamide		766-93-8	13.01	3.54
Phenol	2,3,5-Trimethylphenol		697-82-5	14.19	1.98
Phenol	4-*tert*-Butylphenol		98-54-4	14.71	1.87
PAH	1-Methylnaphthalene		90-12-0	15.10	2.38
Plastic/rubber chemical	Phthalide		87-41-2	15.43	4.45
n-alkanes		nC13	629-50-5	15.61	0.54
Phenol	4-Isopropyl-3-methylphenol		3228-02-2	15.73	1.97
PPCP	Nicotine		54-11-5	16.03	2.16
Phenol	4-*tert*-Butyl-2-methylphenol		98-27-1	16.51	1.79
Degradation product	3,4-fluorobenzamide		85118-04-3	16.57	3.08
OCPs	Tripropyl phosphate	TPP	513-08-6	16.86	1.85
PAH	Biphenyl		92-52-4	16.89	2.42
PAH	2,3-Dimethylnaphthalene		581-40-8	17.72	2.06
n-alkanes		nC14	629-59-4	18.20	0.54
Plastic/rubber chemical	2,4,7,9-Tetramethyl-5-decyne-4,7-diol		126-86-3	18.21	1.05
PAH	2,6-Dimethylnaphthalene		581-42-0	18.52	3.00
PAH	Acenaphthylene		208-96-8	18.52	3.00
PPCP	Isoeugenol		97-53-0	18.64	2.325
Plastic/rubber chemical	Methylparaben		99-76-3	18.91	2.8425
Plastic/rubber chemical	Phthalimide		85-41-6	18.98	3.89
Phenol	4-*n*-Pentylphenol		14938-35-3	19.19	1.7914286
PAH	Acenaphthene		83-32-9	19.36	2.84
Phenol	2,6-Di-*tert*-butyl-4-methylphenol		128-37-0	20.43	1.38

n-alkanes		nC15	629-62-9	20.69	0.54
PAH	Dibenzofuran		132-61-91	20.88	2.43
Plastic/rubber chemical	3-Methyl-2(3H)-Benzothiazolone		2786-62-1	21.21	4.26
Phenol	4-Hexylphenol		2446-69-7	21.50	1.86
PAH	2,3,5-Trimethylnaphthalene		2245-38-7	21.56	2.18
PAH	Fluorene		86-73-7	21.75	2.856
Phthalates	Diethyl phthalate	DEP	84-66-2	21.84	2.81
Plastic/rubber chemical	2-(Methylthio)benzothiazole		615-22-5	22.17	3.46
Phenol	4-*tert*-Octylphenol		140-66-9	22.60	1.75
n-alkanes		nC16	544-76-3	23.05	0.59
OCPs	Tributyl phosphate	TBP	126-73-8	23.31	1.68
PPCP	Benzophenone		119-61-9	23.44	2.83
Phenol	4-Heptylphenol		1987-50-4	23.90	1.8
PAH	1-Methylfluorene		1730-37-6	25.00	2.51
OCPs	Tris-(2-chloroethyl)phospate	TCEP	115-96-8	25.18	3.81
n -alkanes		nC17	629-78-7	25.26	0.60
PPCP	Atrazine		1912-24-9	25.39	3.03
alk	Pristane		1921-70-6	25.47	0.50
Pesticide	Hexachlorobenzene	HCB	118-74-1	25.50	1.89
PAH	Dibenzothiophene		132-65-0	25.51	3.45
PAH	1,2,5,6-Tetramethylnaphthalene		2131-43-3	25.56	2.25
Plastic/rubber chemical	*n*-Butylbenzenesulfonamide		3622-84-2	25.71	3.81
Phenol	4-*n*-Octylphenol		1806-26-4	26.11	1.8
NSO	Benzo[*h*]quinoline		230-27-3	26.19	3.82
PAH	Phenanthrene		85-01-8	26.19	3.46
PPCP	Caffeine		58-08-2	27.06	4.81
n-alkanes		nC18	593-45-3	27.34	0.61
alk	Phytane		638-36-8	27.65	0.54
NSO	Xanthone		90-47-1	27.70	3.73
PAH	4-Methyldibenzothiophene		7372-88-5	27.77	3.14
PCBs	2,4,4’-Trichlorobiphenyl	PCB-28	7012-37-5	28.03	2.67
Phenol	4-*n*-Nonylphenol		104-40-5	28.81	1.62
arom	1-Phenyldodecane		123-01-3	29.26	1.08

PAH	1-Methylphenanthrene		832-69-9	29.39	2.69
n-alkanes		nC19	629-92-5	29.40	0.63
Aromatic	*o*-Terphenyl		84-15-1	29.41	2.45
PCBs	2,2’,5,5’-Tetrachlorobiphenyl	PCB-52	35693-99-3	29.55	2.62
Phthalates	Di-*n*-butylphthalate	DBB	84-74-2	29.62	2.46
PAH	3,6-dimethylphenanthrene		1576-67-6	30.64	3.06
	5@Androstane		438-22-2	31.15	1.60
n-alkanes		nC20	112-95-8	31.32	0.66
PAH	Fluoranthene		206-44-0	31.60	3.80
PPCP	Allethrin		584-79-2	32.06	1.90
PAH	1,2-Dimethylphenanthrene		20291-79-2	32.12	3.37
PPCP	Triclosan		3380-34-5	32.45	3.00
PAH	Pyrene		129-00-0	32.48	4.17
PPCP	Fluconazole		86386-73-4	32.53	4.092
PCBs	2,2’,4,5,5’-Pentachlorobiphenyl	PCB-101	37680-73-2	32.72	2.68
n-alkanes		nC21	629-94-7	33.13	0.66
Plastic/rubber chemical	Bisphenol A	BPA	80-05-7	33.36	4.50
PAH	2,6,9-Trimethylphenanthrene		66271-32-7	33.51	2.86
PBDEs	2,4,4'-Tribromodiphenyl ether	BDE-28	41318-75-6	34.87	3.68
PCBs	2,3’,4,4’,5-Pentachlorobiphenyl	PCB-118	31508-00-6	34.88	2.90
n-alkanes		nC22	629-97-0	34.91	0.70
PAH	1-Methylpyrene		2381-21-7	35.09	4.20
PCBs	2,2’,3,4,4’,5-Hexachlorobiphenyl	PCB-138	35065-28-2	35.82	2.69
Phthalates	Benzyl butyl phthalate	BBP	85-68-7	36.36	3.59
PAH	1,2,6,9-Tetramethylphenanthrene		204256-39-3	36.36	3.15
n-alkanes		nC23	638-67-5	36.63	0.72
PCBs	2,2’,4,4’,5,5’-Hexachlorobiphenyl	PCB-153	35065-27-1	36.68	3.07
OCPs	Triphenyl phosphate	TPhP	115-86-6	37.10	4.37
PAH	Benz[*a*]anthracene		56-55-3	38.17	4.61
n-alkanes		nC24	646-31-1	38.22	0.73
OCPs	Tris(2-ethylhexyl)phosphate	TEHP	78-42-2	38.96	1.14
PBDEs	2,2',4,4'-Tetrabromodiphenyl ether	BDE-47	5436-43-1	39.03	4.13
PCBs	2,2’,3,4,4’,5,5’-Heptachlorobiphenyl	PCB-180	35065-29-3	39.15	2.93

PBDEs	2,3',4',6-Tetrabromodiphenyl ether	BDE-66	189084-62-6	39.60	4.26
n-alkanes		nC25	629-99-2	39.80	0.78
Plastic/rubber chemical	Bumetrizole		3896-11-5	39.83	2.68
PAH	1-Methylchrysene		3351-28-8	40.78	4.58
PPCP	17β-Estradiol	Estradiol	50-28-2	40.88	4.74
n-alkanes		nC26	630-01-3	41.31	0.78
PBDEs	2,2',4,4',6-Pentabromodiphenyl ether	BDE-100	189084-64-8	42.01	4.48
Phthalates	Di-*n*-octyl phthalate	DNOP	117-84-0	42.54	2.16
n-alkanes		nC27	593-49-7	42.75	0.82
PAH	Benzo[*b*]fluoranthene		205-99-2	42.78	5.07
PBDEs	2,2',3,4,4'-Pentabromodiphenyl ether	BDE-85	182346-21-0	42.85	4.66
PAH	Benzo[*e*]pyrene		192-97-2	42.98	4.96
PAH	Benzo[*a*]pyrene		50-32-8	43.01	4.90
n-alkanes		nC28	630-02-4	44.15	0.84
PBDEs	2,2',4,4',5-Pentabromodiphenyl ether	BDE-99	60348-60-9	44.34	5.25
PPCP	Praziquantel		55268-74-1	45.11	5.64
PBDEs	2,2',4,4',5,5'-Hexabromodiphenyl ether	BDE-153	68631-49-2	45.21	5.10
n-alkanes		nC30	638-68-6	46.84	0.91
	17 alpha (H) 21 beta (H)-Hopane		13849-96-2	48.27	2.22
PAH	Benzo[*ghi*]perylene		191-24-2	48.85	0.84
n-alkanes		nC32	544-85-4	49.31	1.04
n-alkanes		nC34	14167-59-0	53.82	1.88
n-alkanes		nC35	630-07-9	55.91	2.33

TOXICITY TESTS

Table S5 Overview of toxicological tests applied to pellet leachates, including species, endpoints, medium and laboratory

**Species**	**Endpoints**	**Medium**	**Lab**
Algae: *Raphidocelis subcapitata*	Growth	Leachate in MiliQ water	University of Gothenburg

Copepod: *Apocyclops royi*	Mortality and behavior	Leachate in artificial seawater	Roskilde University
Fish: *Danio rerio*	Development and locomotion	Leachate in MiliQ water and embryo medium	University of Gothenburg
Primary human cells	Cell viability	Leachate in sterile water	Norwegian Institute of Public Health

Algal growth inhibition test

The experimental design included six exposure treatments. These are: control (no plastic leachate), virgin HDPE 7 d 30 g/L, GP 24 h 10g/L, GP 24 h 30g/L, GP 7 d 10 g/L and GP 7 d 30 g/L. Each treatment was performed in triplicates. The freshwater green microalgal species *Raphidocelis subcapitata* (strain 61.81 from Experimental Phycology and Culture Collection of Algae (EPSAG), Göttingen, Germany) was used in the present study. The assay was performed following the OECD guidelines (test no. 201) with a starting cell density of around 20 000 cells/mL, in 50 mL flasks (NuncTM Non-treated Flasks, ThermoFisher TM) containing 10 mL of 1 BG-11 medium concentrate prepared with the different plastic leachates waters according to the experimental design (see above). The exposure lasted 72h and it was performed in a climate room with a photoperiod of 16/8 h light/dark cycle, with a stable light intensity of 80±5 µmol photons/m2/s and at 20±1 °C. Cultures were constantly agitated using a horizontal shaker set to 55 rpm. As a proxy for growth, chlorophyll fluorescence was measured both at t0 and after 72 h by measuring the fluorescence of a 250 mL aliquots in 96-well plates in a fluorometer (Varioskan Flash version 4.00.53) at the excitation/emission wavelengths 425 nm/680 nm following Gómez-Martínez et al. 2023). Growth inhibition was expressed in percentage and calculated by subtracting the fluorescence at t0 of the unexposed algae from the fluorescence of the exposed algae after 72h and then normalizing to the fluorescence of unexposed algae (control treatment).

Apocyclops royi copepod test

For the copepod exposure, the experimental design included two tests and followed the ISO standard for acute toxicity tests to marine copepods (DS/ISO 14669, 1999). Adult or late-stage copepodids (C5) of *Apocyclops royi* were used in all experiments. The culture originated from aquaculture ponds in Taiwan and has been maintained in continuous laboratory culture at Roskilde University since 2016 (Jepsen et al., 2025). First, a range-finding test was conducted with seven treatments, each containing five *A. royi* individuals and no replicates: a control (0 g L^{-1}) and six concentrations of plastic leachate (0.3, 1, 3, 10, 30, and 100 g L^{-1}). Exposure lasted for 24 and 48 h under a 12:12 h light–dark cycle at 17 °C with continuous oxygen supply. The test volume was 40 mL.

Based on the results of the range-finding test, a definitive test was conducted with seven treatments performed in quadruplicates, each replicate containing five *A. royi* individuals: a control (0 g L^{-1}) and six concentrations of plastic leachate (2, 4, 8, 16, 32, and 64 g L^{-1}). All concentrations were prepared by dilution from a 100 g L^{-1} leachate stock using artificial seawater. The leachate was prepared as described above. Exposure lasted for 24, 48, and 96 h under the same conditions as the range-finding test. In both tests, mortality and sublethal effects (e.g., reduced

activity and low mobility) were assessed under a stereomicroscope. Reduced activity or low mobility was defined as individuals showing swimming behaviour only after gentle stimulation with a dissection needle, in comparison to the control. Validity criteria of DS/ISO 14669 were met using a positive control with 3,5-dichlorophenol as a known toxicant.

Danio rerio fish embryo test (FET) and locomotion test

After leaching, plastic pellets were removed and leachates were stored at -20 ˚C until usage. For the exposure, 20x stock solution was added to leachates and pH was adjusted to 7.0-7.2 to receive zebrafish embryo medium (0.245 g/L $MgSO_4$ x $7H_2O$, 0.021 g/L KH_2PO_4, 0.006 Na_2HPO_4, 0.145 g/L $CaCl_2$ x $2H_2O$, 0.038 g/L KCl, 0.875 g/L NaCl). Zebrafish embryo medium without plastic leachates served as the negative control treatment in the experiment. The following 6 exposure treatments were applied: control (no plastic leachate), virgin HDPE 7 d 30 g/L, GP 24 h 10g/L, GP 24 h 30g/L, GP 7 d 10 g/L and GP 7 d 30 g/L.

Fish embryo exposure and FET

An adjusted version of the OECD 236 guideline (OECD, 2013) was applied in this experiment. Zebrafish (Danio rerio) embryos (AB strain) were received from the Department of Psychiatry and Neurochemistry (Institute of Neuroscience and Physiology, The Sahlgrenska Academy, University of Gothenburg) at ~3h post fertilization (hpf). Fertilization of eggs and identical developmental stage were checked before usage in the experiment under a stereomicroscope. Embryos were kept and exposed at 26 ± 1 °C in a 14 h: 10 h light: dark cycle. The fish embryos were exposed to the control medium and plastic leachates in 6-well plates with 7 embryos per well and 8mL testing solution/well. Each treatment contained 6 replicate wells and a total number of 42 embryos. The embryos were exposed for 96 h with a renewal of solutions every 24 h. Validity criteria of the OECD 236 guideline of a survival of ≥ 90% embryos and ≥ 80% hatching success in the negative control at 96 h were met.

Acute toxicity was assessed by documentation of mortality, morphological abnormalities and hatching in the embryos every 24 h. After 72 h, embryos were moved to 48-well plates to acclimatize for the locomotion assessment at 96 h. After the locomotion tests all embryos were euthanized on ice and stored at -80°C.

Zebrafish larvae locomotion assessment

Locomotion tests were conducted at 96 h exposure in 48-well plates with 1 embryo per well with randomly assigned positions with a total of 40 embryos/treatment where each treatment group was present on each plate (8 embryos/treatment/plate). Five technical replicate plates were run in the ZebraBox (ViewPoint Life Sciences Inc., Montreal, Canada). Locomotion of embryos in the wells was recorded and tracked by the automatic behavior tracking system with ViewPoint® Zebralab software (ViewPoint Life Sciences Inc., Montreal, Canada). A protocol with a total duration of 1 h 5 min of behavior tracking with alternating light (19000 lux) and dark phases of 5 min: 5 min was applied. In 10-second intervals, the number of movements (activity), distance travelled in mm and the duration of movement in seconds were measured. As of the program's definition minor movements consist of 0 – 2.1 mm, small movements of 2.1 – 6.1 mm and large movements of > 6.1 mm distance travelled by the fish. Raw data was analyzed with the software Fish Movement Analyzer (Azora König Kardgar,

https://github.com/azorakk/FishMovementAnalyzer). The total activity, total distance travelled, and total duration of activity including all movement sizes were determined for each fish in all light and dark cycles.

Toxicity tests with Primary Human Cells (Peripheral Blood Mononuclear cells)

Sample Preparation and Cell isolation

Peripheral blood samples were collected from three healthy volunteers in 10 mL ethylenediaminetetraacetic acid (EDTA) Vacutainer® tubes (BD Medical Supplies, Crawley, UK) by a trained member of the research team. Samples were processed within four hours of collection. Peripheral blood mononuclear cells (PBMCs) were isolated using SepMate™-50 (IVD) (STEM CELL™ Technologies, Vancouver, Canada). Briefly, whole blood was diluted with an equal volume of Dulbecco's Phosphate-Buffered Saline (DPBS) (Thermo Fischer Scientific) in a 50ml conical tube (Greiner Bio-One; Frickenhausen, Germany). Lymphoprep™ Density gradient medium (STEM CELL™ Technologies, Vancouver, Canada) was added by pipetting into the central hole of the SepMate™-50 tube. Diluted blood was added from the side into the upper chamber of the SepMate™-50 tube, separated by the insert. The tube was centrifuged at 1200 x g for 10 minutes at Room Temperature (RT) with no brake. The top layer, enriched with PBMCs and plasma was poured off onto a new 50ml conical tube. Enriched PBMCs were washed twice with PBS at 200 x g for 10 minutes at RT without brakes. PBMCs were counted using muse cell analyzer (EMD Millipore Corporation, USA) and were suspended in 1ml of PBS with 1mM EDTA and 1% heat inactivated Human serum (Merck, Saint Louis, USA) in 15ml tube (Sarstedt, Germany).

Cell Viability Assay (PrestoBlue™ HS)

Cell viability assay was performed using PrestoBlue (PB) as per the manufacturer protocol. PBMCs were maintained in RPMI 1640 medium (Thermo Fischer Scientific) supplemented with 10% heat inactivated Human AB serum and 1% antibiotics (100 U/mL penicillin, 100 µg/mL streptomycin). 50k cells were seeded in sterile flat bottom 96 well plates (Sarstedt, Germany). Three different volumes of plastic leachates (10, 20 and 50ul) were tested for different time points (24, 48 and 72 hours). 10ul of PrestoBlue was added into the wells containing 190ul RPMI 1640 medium along with the leachates and cells. The plate was then incubated for different time points (24, 48 and 72 hours) at 37-degree incubator with 5% CO_2. The fluorescence was measured using a CLARIOstar microplate reader (BMG, LABTECH, Germany). with excitation and emission wavelengths set at 545-20 nm and 600-40, respectively. The cell viability was expressed as a percentage relative to unstimulated control.

STATISTICAL ANALYSIS

Statistical differences in algal growth rate inhibition among treatments were assessed by one-way analysis of variance (ANOVA), followed by a Tukey post-hoc test for multiple comparisons using the R package multcompView. In all tests, p-values <0.05 were considered statistically significant. Zebrafish embryo data was statistically analyzed and visualized with GraphPad Prism version 10.2.2 for Windows, GraphPad Software, Boston, Massachusetts USA, www.graphpad.com. First, the data was tested for normality by applying the Shapiro-Wilk test. Then, the statistical differences among groups were assessed by Kruskal-Wallis test, and two-way repeated-measure analysis of variance (ANOVA) and followed by Dunn’s and Tukey's multiple comparisons test respectively. Statistical analysis and visualization of PBMC viability were performed in RStudio

2025.09.1+401. For each combination of leaching time, pellet number, leachate volume, and exposure duration, the donor-level differences were calculated as:

$$\Delta\% = Viability_{exposed}\% - Viability_{vehicle}\%$$

These differences were tested against zero using a two-sided t-test. For each leachate volume, p-values were adjusted across all three time points using the Benjamini–Hochberg procedure. Significant differences are indicated by * and **, corresponding to adjusted p-values < 0.05 and < 0.01, respectively.

Table S6. Mean concentrations of the targeted plastic additives in pellets collected from Illa d'Arousa (Galicia, NW Spain), determined in duplicates and expressed in ng g^{-1}. LOQ: limit of quantification of the analytical method.

Compound name	Category*	LOQ	Concentration
UV-328	UV stabilizer	0.16	1.07
UV-3638	UV stabilizer	100.00	7465.51
2,2',6,6'-Tetrabromobisphenol A	Flame retardant	4.00	73.53
2-Mercaptobenzothiazole	Antioxidant	4.00	<LOQ
Benzoic Acid	Antimicrobial	100.00	454.77
Benzophenone	UV stabilizer	0.80	4.45
Benzyl butyl phthalate	Phthalate	0.16	11.49
Bis(2-ethylhexyl) phthalate	Phthalate	0.80	14.00
Dibutyl phthalate	Phthalate	4.00	116.38
Didecyldimethylammonium chloride	Antimicrobial	0.008	24.17
Dihexyl phthalate	Phthalate	0.16	0.26
Dioctyl phthalate	Phthalate	0.16	15.39
Disulfiram	Pharmaceutical	4.00	7162.80
Phthalic acid anhydride	Phthalate (degradation product)	4.00	220.50
Triphenyl phosphate	Flame retardant	0.03	22.65
Di(2-ethylhexyl)tetrabromophthalate	Phthalate	100.00	11198.19
Dibenzylamin	Catalyst	0.006	0.10
Oleamide	Lubricant	0.80	2.88
Tinuvin 770	UV stabilizer	0.80	6.07
Tinuvin RS	UV stabilizer	0.16	0.61
Cyasorb UV-531	UV stabilizer	0.80	21.84
Tinuvin P	UV stabilizer	4.00	64.56
Bis(2,4-di-tert-butylphenyl) phosphate	Antioxidant	0.03	1.15
Irganox 1010	Antioxidant	0.16	51.83
Irganox 1076	Antioxidant	60.00	75200.00
2,4-di-tert-butylphenol (2,4-DTBP)	Antioxidant	5.00	750.00

Taris (2,4-di-tert-butylphenyl) phosphate (TDTBPP)	Antioxidant	70.00	113000.00

*Some compounds have more than one function and the main one is listed here.

Table S7. Mean concentrations of target plastic additive chemicals measured in the ASW leachates from pellets collected from the Illa D´Arousa (Galicia, NW Spain), determined in duplicates and expressed in µg L-1. LOQ: limit of quantification of the analytical method (Galicia, NW Spain) in µg L-1. LOQ: limit of quantification of the analytical method

Compound name	Category	LOQ	Concentration
Benzyl butyl phthalate	Phthalate	1.60	59.37
Benzyldimethylamine	Amine	8.00	44.37
Bis(2-ethylhexyl) phthalate	Phthalate	8.00	224.97
Bisphenol S	Bisphenol	1.60	1.69
Didecyldimethylammonium chloride	Antimicrobial	8.00	19.03
Dihexyl phthalate	Phthalate	1.60	2.99
Diisoheptyl phthalate	Phthalate	1.60	4.97
Dimethoxyethyl phthalate	Phthalate	1.60	4.19
Di-n-propyl-phthalate	Phthalate	0.32	2.86
Dioctyl phthalate	Phthalate	1.60	206.12
Phthalic acid anhydride	Phthalate (degradation product)	40.00	503.26
Propyl 4-hydroxybenzoate	Paraben (preservative)	1.60	3.07
Triphenyl phosphate	Flame retardant	0.32	27.41
Tris(2-chloroethyl) phosphate	Flame retardant	8.00	8.68
Dibenzylamin	Amine	0.06	0.27
Tinuvin 770	UV stabilizer	8.00	17.06
Tinuvin RS	UV stabilizer	1.60	5.68
Cyasorb UV-531	UV stabilizer	8.00	18.21
Irganox 1010	Antioxidant	1.60	2.84
Tripropylphosphate	Flame retardant	0.32	0.52
Tinuvin 249	UV stabilizer	0.32	0.62

Table S8. Morphological assessment of zebrafish embryos after 24, 48, 72 and 96 h exposure to control (no plastic leachate), virgin HDPE or Galician spill PE pellet leachates (24 h, 7d and 10 g/L, 30 g/L). Mortality and malformations are noted as the total number of embryos, hatching rate in average (±SD) percentage of the total number of embryos (n = 42). Mortality and malformation numbers and hatching rates did not significantly differ compared to the control ($p > 0.05$). Malformations: □ = short tail, ∆ = less pigmentation.

		Control	vi. HDPE 7 d 30 g L^{-1}	Galician PE 24 h 10 g L^{-1}	Galician PE 24 h 30 g L^{-1}	Galician PE 7 d 10 g L^{-1}	Galician PE 7 d 30 g L^{-1}
24 h	Mortality	0	0	0	0	2	1
	Malformations	0	0	0	0	0	0
	Hatching (%)	0	0	0	0	0	0
48 h	Mortality	0	0	0	0	0	0
	Malformations	0	0	1□	0	3∆	5∆
	Hatching (%)	0	0	0	0	0	0
72 h	Mortality	0	0	0	0	0	0
	Malformations	0	0	1□	0	0	0
	Hatching (%)	71 ± 14	52 ± 24	95 ± 11	53 ± 23	83 ± 15	100 ± 0
96 h	Mortality	0	0	0	0	0	0
	Malformations	0	0	1□	0	0	0
	Hatching (%)	100 ± 0	100 ± 0	100 ± 0	100 ± 0	100 ± 0	100 ± 0

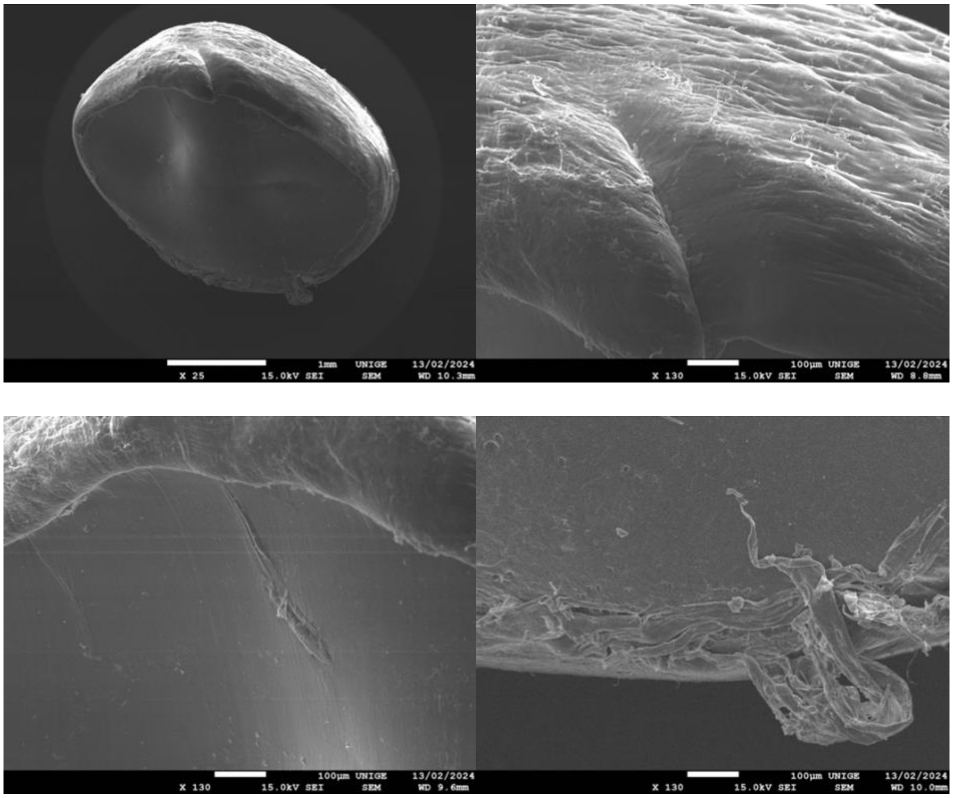

Figure S3 SEM images of a representative PE pellet collected at Arousa, at different magnifications.

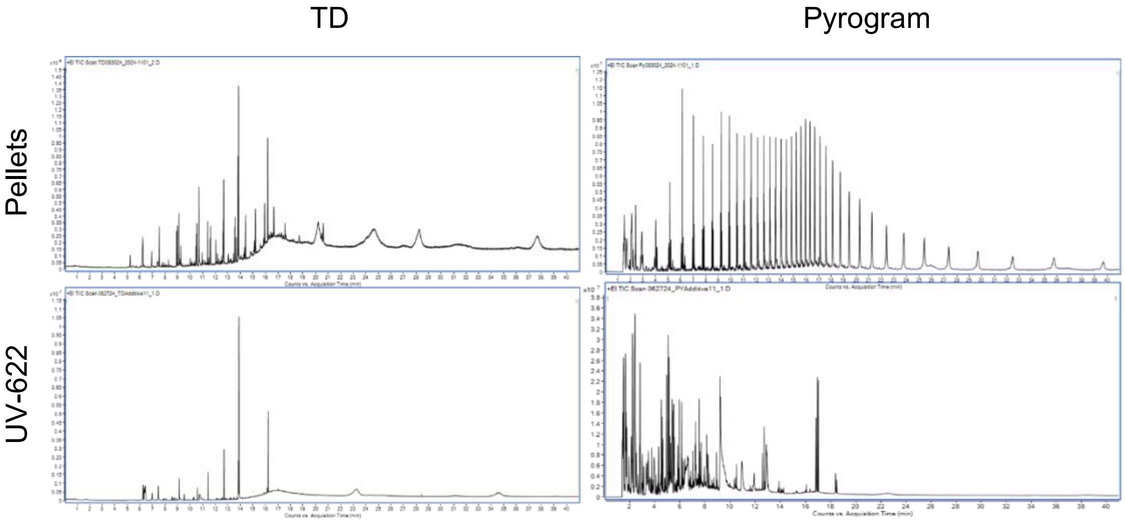

Figure S4. Thermal desorption chromatogram (top left) and pyrogram (top right) of pellets. Thermal desorption chromatogram (bottom left) and pyrogram (bottom right) of pure Tinuvin UV-622 stabilizer.

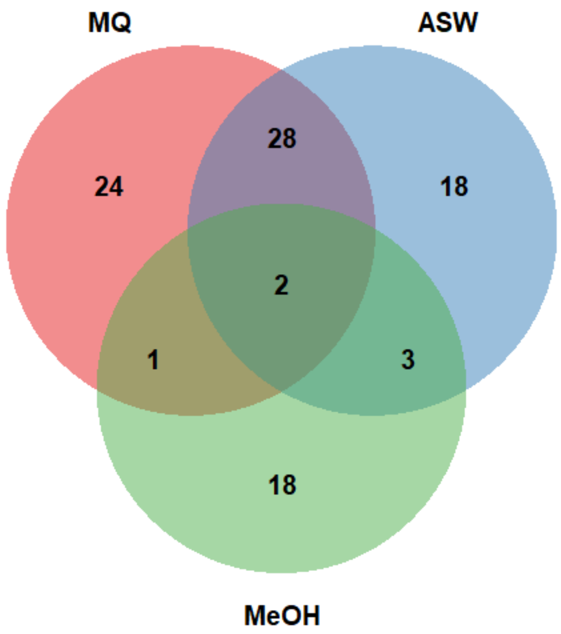

Figure S5. Number and distribution of detected peaks from GC×GC-MS analysis of the pellet MeOH extract and of MQ and ASW leachates (7 days) where tentative identities could be assigned.

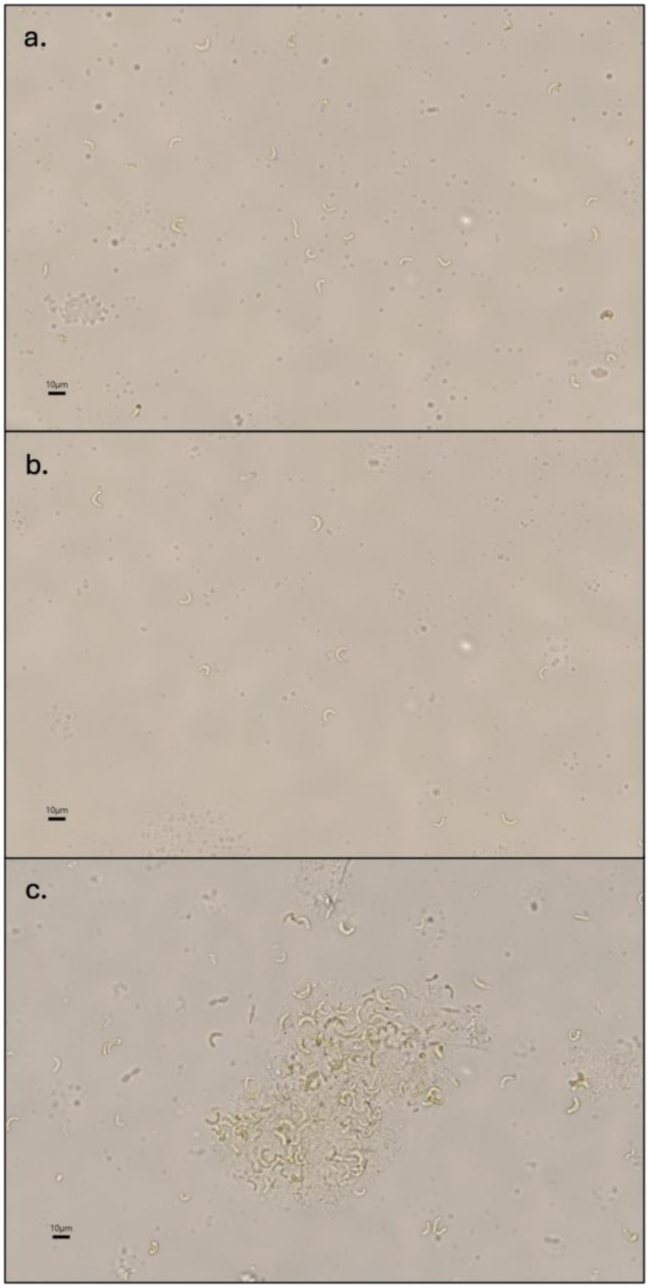

Figure S6. Light microscopy images at 40X magnification, of the control algae (a), algae exposed to virgin HDPE pellet leachate (b), and algae exposed to 7 days, 30 g L-1 Galician pellet leachates (c). The scale bar in each picture corresponds to 10 µm .

SUPPLEMENTARY REFERENCES

- Danish Standard Foundation. 1999. ISO 14669:1999(E). Water quality - determination of acute lethal toxicity to marine Copepods (Copepoda, Crustacea). Copenhagen, Denmark: Danish Standard Foundation.
- Jepsen, P. M., Dinsen, C. H., Øllgaard, E. S. H., Jedal, J. Y. B., Aggerholm, L., Salomonsen, T., & Ramløv, H. (2025). Coping with salinity change: How does the cyclopoid copepod Apocyclops royi (Lindberg 1940) do it? Comparative Biochemistry and Physiology Part A: Molecular & Integrative Physiology, 301, 111794. https://doi.org/https://doi.org/10.1016/j.cbpa.2024.111794
- R Development Core Team. *R: A language and environment for statistical computing*. R Foundation for Statistical Computing. http://www.R-project.org.
- Sørensen, L.; Gomes, T.; Igartua, A.; Lyngstad, I. L.; Almeida, A. C.; Wagner, M.; Booth, A. M. Organic Chemicals Associated with Rubber Are More Toxic to Marine Algae and Bacteria than Those of Thermoplastics. *Journal of Hazardous Materials* **2023**, 131810. https://doi.org/10.1016/j.jhazmat.2023.131810.
- Sørensen, L.; Schaufelberger, S.; Igartua, A.; Størseth, T. R.; Øverjordet, I. B. Non-Target and Suspect Screening Reveal Complex Pattern of Contamination in Arctic Marine Zooplankton. *Science of The Total Environment* **2023**, *864*, 161056. https://doi.org/10.1016/j.scitotenv.2022.161056.
- van Den Dool, H.; Kratz, P. Dec. A Generalization of the Retention Index System Including Linear Temperature Programmed Gas—Liquid Partition Chromatography. *Journal of Chromatography A* **1963**, *11*, 463–471. https://doi.org/10.1016/S0021-9673(01)80947-X.
